# Effect of applying oyster shell powder on soil properties and microbial diversity in the acidified soils of pomelo garden

**DOI:** 10.1186/s40793-025-00721-6

**Published:** 2025-05-24

**Authors:** Yuanyuan Li, Qiong Zhang, Lixia Zhu, Jing Yang, Jingjing Wei, Yunhe Li, Xiaohuang Chen

**Affiliations:** 1 Provincial Key Laboratory of Landscape Plants with Fujian and Taiwan Characteristics, Minnan Normal University, Zhangzhou, 363000 China; 2 Fujian Province University Key Laboratory of Pollution Monitoring and Control, Minnan Normal University, Zhangzhou, 36300 China; 3 Fujian Province Key Laboratory of Modern Analytical Science and Separation Technology, Minnan Normal University, Zhangzhou, 363000 China

**Keywords:** Oyster shell, Bacteria, Fungi, Pomelo, Soil acidification

## Abstract

The application of oyster shell has recently been used to increase soil pH in Southern China. However, little is known about causal shifts in the rhizosphere microbial community of pomelo trees, especially in orchards that have experienced natural accumulation of heavy metals over many years due to continuous fertilization and soil acidification. This study evaluated the effects of oyster shell powder applied for 1 year (T1), 2 years (T2) and 3 years (T3), alongside a control group with no soil amendments (Control; CK), on soil acidification and microbial diversity. Our findings demonstrated that the application of oyster shell significantly increased soil pH and reduced the concentrations of heavy metals such as thallium (Tl), chromium (Cr), and manganese (Mn). Illumina sequencing-based community analysis revealed that oyster shell application significantly increased the alpha diversity indices of both bacterial and fungal communities and influenced their distribution in the soil. Notably, all oyster shell-treated groups (T1-T3) showed significantly higher relative abundances of beneficial microbes (e.g., *Nitrolancea*,* Vicinamibacterales*) and those involved in carbohydrate degradation and nitrogen fixation compared to the control. Conversely, the relative abundances of *Acidibacter* and *Chujaibacter* (associated with heavy metal degradation and soil-borne diseases), *Trichoderma* and *Acremonium* (plant-beneficial fungi), as well as functionally annotated groups linked to nitrogen assimilation and pathotrophic modes (predicted via FUNGuild analysis), decreased significantly. Our results suggest that the application of oyster shell powder amendments contributes to improved soil properties and microbial environments; however, the effects on soil nitrogen cycling and fungal function are complex, warranting further research.

**Supplementary Information:**

The online version contains supplementary material available at 10.1186/s40793-025-00721-6.

## Introduction

Citrus fruit represent one of the most important agricultural products globally, accounting for 20–30% of the total fruit industry. As the world’s largest producer of citrus, China boasts a rich germplasm resource that includes citrus, orange and pomelo, which are extensively cultivated in the southern region of the Yangtze River. Fujian province, located in southeastern China, has the highest honey pomelo production and the greatest output value. However, excessive fertilization has accelerated soil acidification in pomelo plantations, with Fujian orchards showing severe acidification (pH < 5) in 90% of soils—below pomelo’s minimum growth threshold. Soil acidification undermines the soil’s capacity to retain nutrients, resulting in the rapid loss of alkaline cations and soil organic matter (SOM). Furthermore, acidification has led to increased mobility of heavy metals (iron (Fe), manganese (Mn), copper‌ (Cu), lead (‌Pb), zinc (Zn), and aluminum (Al)) in the soil [1]. This process has consequently resulted in nutrient imbalance, decreased yield and quality, and a series of secondary environmental risks associated with honey pomelo cultivation. Therefore, the application of effective soil conditioners is essential for ameliorating acidic soils [2].

Oysters are among the most economically important shellfish species along coastal areas. Fujian Province is a leading producer of oysters in China, however, the disposal of oyster shells has emerged as a significant environmental concern. Oyster shells, primarily composed of calcium carbonate (CaCO_3_), can neutralize soil acidity, improve permeability, and mitigate heavy metal toxicity to enhance plant growth [3]. Oyster shell powder also contains organic matter (e.g., chitin derivatives) and trace minerals that support microbial growth by serving as carbon sources and enzyme cofactors [4]. This makes it an effective, eco-friendly soil amendment from biomaterials [5]. Recent studies have shown that calcined oyster shell powers significantly reduced the acidification and enhanced the quality of soils used for crops [3], vegetables [5, 6] and fruits [7, 8]. While the effects of oyster shells on microorganisms in acidic soils have also been investigated, findings have been inconsistent. For instance, Huang et al. [9] found that oyster shell treatment had no discernible effect on the structure of the bacterial community in contaminated and acidified pot soils. Additionally, the diversity of inter-root soil bacteria in tomato plants modestly decreased when treated with oyster shell soil conditioners derived from acidic red clay soil [6]. Conversely, Zhao et al. [10] reported that oyster shell powder significantly improved the abundance and diversity of bacterial community in acidified soils of sweet tea gardens. Jiang et al. [11] proved that oyster shell powder enhances rhizospheric microbial-mediated suppression of root-knot nematodes. Given these mixed results, we note that existing research on the impact of oyster shells on microbial communities—particularly fungal communities—in fruit orchards is limited and fragmented, with even fewer studies addressing the functional aspects of microbial communities.

In this study, the field trials of three years were conducted on the acidic soils of a long-established pomelo plantation. The properties and enzyme activity of the soil, as well as the structure and diversity of soil microbial communities were assessed under different application durations of oyster shell. We are trying to elucidate that: (1) the impacts of oyster shell application on both soil properties and soil micro-ecology in acidic pomelo cultivation; (2) the differentiated responses between bacterial and fungal communities to oyster shell application. This research aims to enhance our empirical understanding of microbial ecology and to facilitate the development of oyster shell-based soil amendments.

## Materials and methods

### Site description, field trial description and sample collection

The experimental site was located at the honey pomelo plantation in Yiling, Pinghe county, Fujian (24°22′6′′N, 117°19′21′′E). The soils are classified as Typic Hapludults under the USDA Soil Taxonomy system (equivalent to Acrisols in the WRB system). Notably, long-term pomelo orchard management has been reported to accelerate topsoil acidification [12]. Additionally, this area is characterized by a subtropical monsoon climate, with an average temperature of 24 °C. In 2021, the average temperature was the highest recorded, while the summers of 2022 and 2023 were slightly cooler. Annual precipitation in 2021 totaled 1041 mm, which was 22% and 23% higher than that in 2022 and 2023, respectively. Meteorological conditions are shown in Fig. S1. The soils at the site were classified as acidic (pH < 5.6) or strongly acidic (pH < 4.6), exhibiting reduced levels of organic matter and essential cations, including calcium (Ca), Zn and magnesium (Mg). Additionally, the levels of available phosphorus and potassium were found to be lower than those typically observed in standard soils [13].

**Fig. 1:**
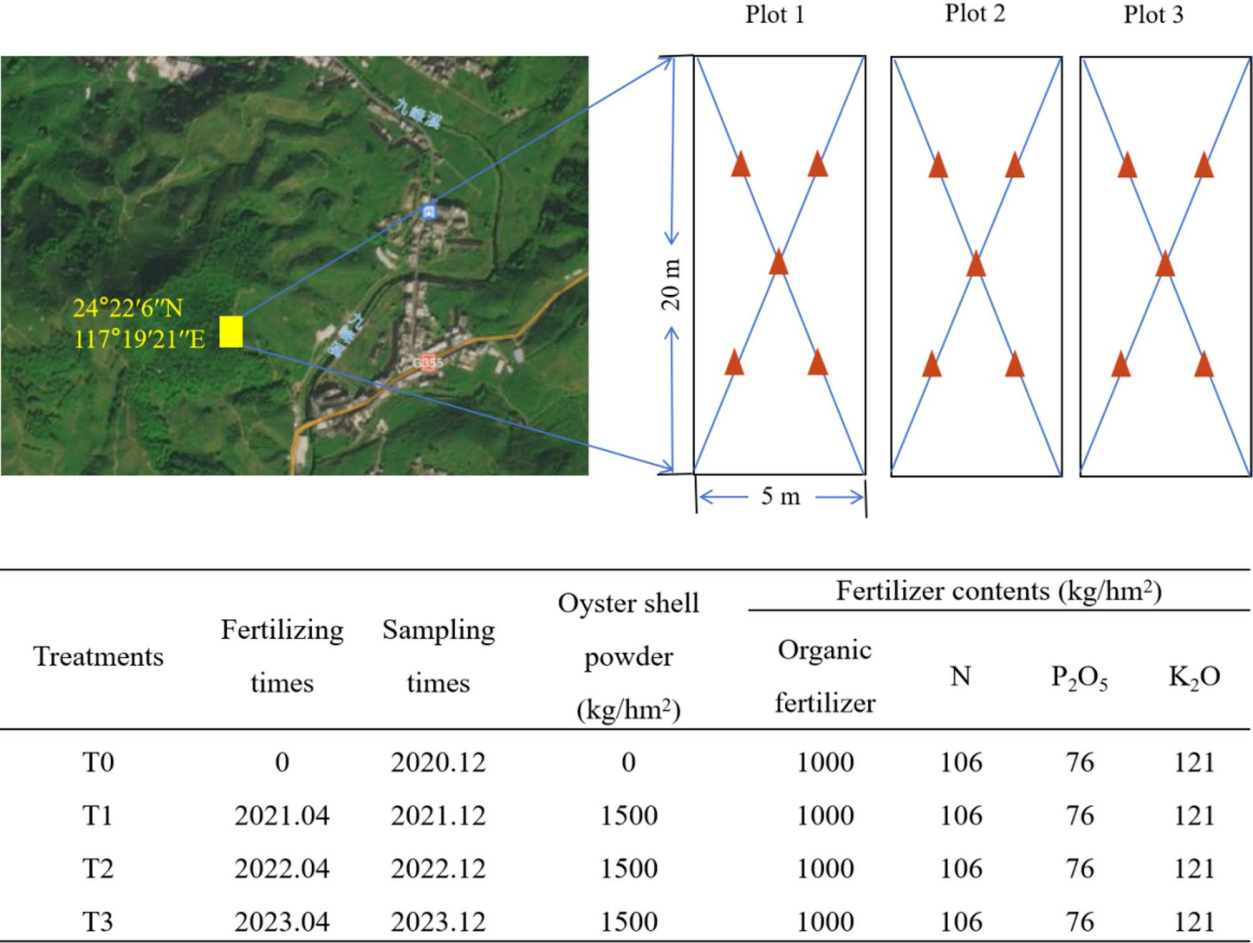
Geographical locations of sampling site and field trial description. The red triangle denoted sampling point in each experimental plot. Totally five samples of soils from one plot were thoroughly mixed to form one composite sample

This study was conducted over a four-year field trial period, commencing in April 2020. Based on prior investigations, pomelo planting areas with soils at a pH of 4.5 were selected as the test sites. Each pomelo plantation plot measured 20 m × 5 m, with a total of three field trial plots established as biological replicates (Fig. 1). The fields received base fertilization consisting of 360 kg·ha^− 1^ of a balanced fertilizer (N-P_2_O_5_-K_2_O, 17-17-17) and 1000 kg·ha^− 1^ of organic manure, with an additional 300 kg·ha^− 1^ of fertilizer (N-P_2_O_5_-K_2_O, 15-5-20) applied after flowering. Beginning in April 2021 through 2023‌, calcined oyster shell powder fertilizer was applied annually at 1,500 kg·ha⁻¹. This application rate was selected based on optimal thresholds identified in previous agricultural studies: research in Fujian melon orchards [14] and Jiangxi rice fields [15] both demonstrated that 1,500-2,250 kg·ha⁻¹ most effectively alleviated soil acidification while improving crop yields. To balance effectiveness with farmers’ economic costs, we adopted the lower threshold of 1,500 kg·ha⁻¹, which was further validated by recent trials in Fujian grape orchards showing maximum yield improvements at this rate [16]. ‌Alongside this treatment‌, standardized protocols for fertilization, irrigation, and soil disinfection were uniformly implemented and monitored throughout the experimental period.

Soil samples were collected annually from 2020 to 2023 (Fig. 1). The experiment was conducted across ‌three independent field plots (biological replicates)‌. The control sample (CK) represents the control group without oyster shell powder application, sampled in 2020. Subsequently, ‌T1, T2, and T3 treatments‌ were applied sequentially to ‌separate plots‌ for one, two, and three years, respectively, with sampling completed in 2021, 2022, and 2023. This design ensures that each treatment duration corresponds to a distinct biological replicate plot, avoiding temporal confounding. Soil sampling was collected following the method described by Christel et al. [17]. Approximately, 1 kg of soil was collected from each site at a depth of 15 cm using a diagonal sampling method in November of each year. Immediately upon collection, the soil samples were placed in an ice box. In the laboratory, the samples were sieved through a 10 mm mesh to remove plant residues and subsequently stored at -20 °C for enzyme activity assays and DNA extraction. Additional sub-samples were dried at room temperature for physical and chemical property determination.

### Physical and chemical properties determining

Soil pH was measured in accordance with the NY/T1377-2007 protocol. Soil electrical conductivity was determined using a conductivity meter (DDBJ-350, Shanghai). Cation exchange capacity (CEC) was determined using the neutral ammonium acetate method [18]. Ammonium nitrogen (AN) and available potassium (AK) contents were quantified using a spectrophotometer (ZX11-UV-1000, Beijing) and a flame photometer (FP6400, Shanghai), respectively. The readily oxidizable carbon (ROC) content was calculated employing the oxidation-reduction method [19]. Enzymatic activities of soil dehydrogenase (DHA), sucrase (SC) and urease (UR) were measured following the protocols provided by Suzhou Keming Biotechnology Co., Ltd. The concentrations of metal elements, including Mg, Fe, Cr, Mn, Tl, cobalt (Co), and nickel (Ni), were determined using inductively coupled plasma mass spectrometry (ICP-MS). All parameter measurements were conducted in triplicate to ensure biological and analytical replicability.

### Soil DNA extraction and molecular analysis of soil microorganisms

Genomic DNA from a total of 12 soil samples was extracted using the TransGen AP221-02 Kit. The V4 region of bacterial 16S rRNA gene was amplified with the primers 515F (5’-GTGCCAGCMGCCGCGGTAA-3’) and 806R (5’-GGACTACHVHHHTWTCTAAT-3’) [20]. The V4 region of fungal 18 S rRNA gene was amplified using the primers SSU0817F (5’-TTAGCATGGAATAATRRAATAGGA-3’) and 1536R (5’-TCTGGACCTGGTGAGTTTCC-3’) [21]. The PCR reaction mixture included: TransStart FastPfu, 4 µL; 2.5 mM dNTPs, 2 µL; forward primer (5 µM), 0.8 µL; reverse primer (5 µM), 0.8 µL; FastPfu Polymerase, 0.4 µL; bovine serum albumin, 0.2 µL; template DNA, 10 ng; then, added the distilled water to 20 µL. The amplification procedure was as follows: initial denaturation at 95 ℃ for 3 min, followed by 27 cycles of denaturing at 95 ℃ for 30 s, annealing at 55 ℃ for 30 s and extension at 72 ℃for 45 s, and single extension at 72 ℃ for 10 min, and end at 4 ℃. The PCR product was extracted from 2% agarose gel and purified using the PCR Clean-Up Kit (YuHua, Shanghai, China) according to manufacturer’s instructions and quantified using Qubit 4.0 (Thermo Fisher Scientific, USA). Purified amplicons were pooled in equimolar amounts and paired-end sequenced on an Illumina Nextseq2000 platform (Illumina, San Diego, USA) according to the standard protocols by Majorbio Bio-Pharm Technology Co. Ltd. (Shanghai, China). The obtained sequences underwent quality control and filtering with fastp (v0.19.6), followed by merging and denosing using FLASH (v1.2.11) and DADA2. Briefly, the quality filtering parameters were set as follows: (1) Filter reads with tail quality values were below 20 and a 50 bp window was set simultaneously. If the average quality value within the window was below 20, the back end bases from the window was cut off. Reads with quality control values below 50 bp was filtered and reads containing N bases were removed; (2) According to the overlap relationship between paired end reads, paired reads were merged into a sequence with a minimum overlap length of 10 bp; (3) The maximum allowed mismatch ratio in the overlap area of the concatenated sequence was 0.2, and non matching sequences were screened; (4) Samples based on the barcode and primers at the beginning and end of the sequence were distinguished, and the sequence direction was adjusted. The barcode allowed 0 mismatches, and the maximum primer mismatch was 2. A total of 583,599 sequences were obtained from the bacterial community, yielding an average of 48,633 sequences per sample, while 344,738 sequences were retrieved from the fungal community, with an average of 28,728 sequences per sample (Table S1). As indicated by rarefaction curves, all microorganisms in the samples were sufficiently covered (Fig. S2). The sequences after DADA2 processing were commonly referred to as Amplicon Sequence Variants (ASVs). To minimize the impact of sequencing depth on subsequent Alpha and Beta diversity analysis, all sample sequences were flattened to 20,000. After flattening, the average sequence coverage (Good’s coverage) of each sample were reached 99.09%. Qualified sequences were subsequently mapped to the Sliva 16 S rRNA gene database (v138) for bacterial species identification and to the ‌NCBI Nucleotide Database (v20221012) [22, 23] for fungal species identification using the Naive Bayes classifier in QIIME2 [24]. Then, we used the Functional Annotation of Prokaryotic Taxonomy (FAPROTAX) database to analyze the functional groups of bacteria in the soil with default settings in the output functional Table [25]. Comparisons of fungal functional groups were conducted using Python 3.7 in conjunction with the FUNGuild database [26].

### Statistical analysis

The software Mothur (1.30) was used to analyse the alpha diversity indices, including Chao, Ace, Shannon and Simpson (Table S2). Statistical comparisons among groups were conducted using the Least Significant Difference (LSD) t-test to identify significant differences. The differences in microbial community structure (beta diversity) among groups were statistically assessed through Principal Coordinates Analysis (PCoA) and Permutational Multivariate Analysis of Variance (PERMANOVA), utilizing R software (3.3.1, vegan package) [27]. Additionally, the UPGMA algorithm was used to calculate Bray-Curtis distances and perform hierarchical clustering analysis [28]. The relative abundances of bacterial and fungal groups, as well as the frequencies of functional genes, were compared using *p*-values derived from the Kruskal-Wallis H test, analyzed with SPSS software (26.0). Mantel tests were used to determine the effects of biotic and abiotic factors on microbial diversity [29]. Further, Pearson’s correlation coefficients were calculated to reveal the relationships among soil physico-chemical parameters, enzyme activities with both the bacterial and fungal diversity indices. The data used in network construction conformed to the absolute value of Pearson’s rank correlation coefficient > 0.7 and *p* < 0.05 in the correlation analysis, and the network plots were generated by using Gephi (0.9.2).

## Results

### Soil properties and enzyme activities

The soil properties exhibited significant variations over the course of the study (Table 1). Soil pH significantly decreased in T1 but increased significantly to 5.58 from T2 to T3. The maximum concentration of NH_4_^+^ was observed in T1. Soil EC and the content of AK initially decreased from T1 to T2, subsequently increasing significantly in T3 following the application of oyster shell powders. The contents of CEC and ROC also increased significantly over the application period. In contrast, the concentrations of metals (including Cr, Mn, Fe and Tl) significantly decreased from T1 to T3 with the application of oyster shell powders. Notably, Mg content decreased significantly after the application of oyster shell powders and exhibited only slight changes across different application years. The activities of SDH and UR significantly increased with prolonged application of oyster shell powders. The activity of soil SC significantly decreased in T1 and T2 compared to the control but then significantly increased in T3, returning to baseline levels. These results indicated that the application of oyster shell powders could have significantly influenced soil fertility and mitigated heavy metal contamination.

**Table 1:** Soil properties under different years of oyster shell powder application in pomelo gardens

Properties	CK	T1	T2	T3
pH	4.50 ± 0.09^b^	4.33 ± 0.05^a^	4.66 ± 0.08^b^	5.58 ± 0.11^c^
EC (µs.cm^− 1^)	111.23 ± 4.56^c^	100.23 ± 4.74^b^	37.63 ± 2.16^a^	172.83 ± 4.38^d^
CEC (cmol.kg^− 1^)	13.29 ± 0.21^a^	13.43 ± 0.07^a^	13.44 ± 0.33^a^	14.05 ± 0.25^b^
AK (mg.kg^− 1^)	86.47 ± 0.70^a^	374.82 ± 23.99^c^	62.68 ± 3.33^a^	277.36 ± 20.35^b^
NH_4_^+ (mg.g−1)^	2.72 ± 0.01^a^	14.70 ± 1.61^b^	0.78 ± 0.02^a^	1.95 ± 0.13^a^
ROC ^(mg.g−1)^	4.05 ± 0.05^a^	4.91 ± 0.60^b^	5.56 ± 0.18^b^	7.49 ± 0.59^c^
Mg (mg.kg^− 1^)	0.30 ± 0.15^b^	0.16 ± 0.06^a^	0.16 ± 0.02^a^	0.08 ± 0.03^a^
Cr (mg.kg^− 1^)	19.55 ± 1.72^c^	13.82 ± 2.59^b^	7.18 ± 0.74^a^	10.36 ± 1.63^a^
Mn (mg.kg^− 1^)	140.57 ± 19.83^b^	92.95 ± 9.50^a^	82.05 ± 2.63^a^	70.85 ± 0.18^a^
Fe (mg.kg^− 1^)	161.57 ± 6.55^b^	185.13 ± 5.73^c^	104.24 ± 5.93^a^	98.18 ± 11.81^a^
Co (mg.kg^− 1^)	1.66 ± 0.05^ab^	1.99 ± 0.09^b^	1.41 ± 0.03^a^	1.43 ± 0.33^a^
Ni (mg.kg^− 1^)	6.10 ± 0.44^c^	4.63 ± 0.70^b^	2.42 ± 0.12^a^	5.79 ± 0.31^c^
Tl (mg.kg^− 1^)	0.39 ± 0.01^b^	0.36 ± 0.01^ab^	0.36 ± 0.00^ab^	0.33 ± 0.04^a^
SDH (µg·d^− 1^·g^− 1^)	6.68 ± 0.26^a^	10.88 ± 1.21^a^	11.25 ± 1.07^a^	23.75 ± 7.48^b^
SC (µg·d^− 1^·g^− 1^)	4.00 ± 1.14^b^	1.81 ± 0.53^a^	0.07 ± 0.01^a^	3.91 ± 0.48^b^
UR (µg·d^− 1^·g^− 1^)	421.31 ± 27.73^a^	355.02 ± 70.61^a^	543.27 ± 37.31^a^	4328.16 ± 191.58^b^

Note: CK indicated soils without oyster shell powder application; T1, T2 and T3 represented soils with oyster shell powder application for one year, two years and three years, respectively. Data in the table were presented as mean ± standard error (SE). Different lowercase indicated significant differences at the *P* < 0.05 level

### Bacterial and fungal diversity

**Fig. 2:**
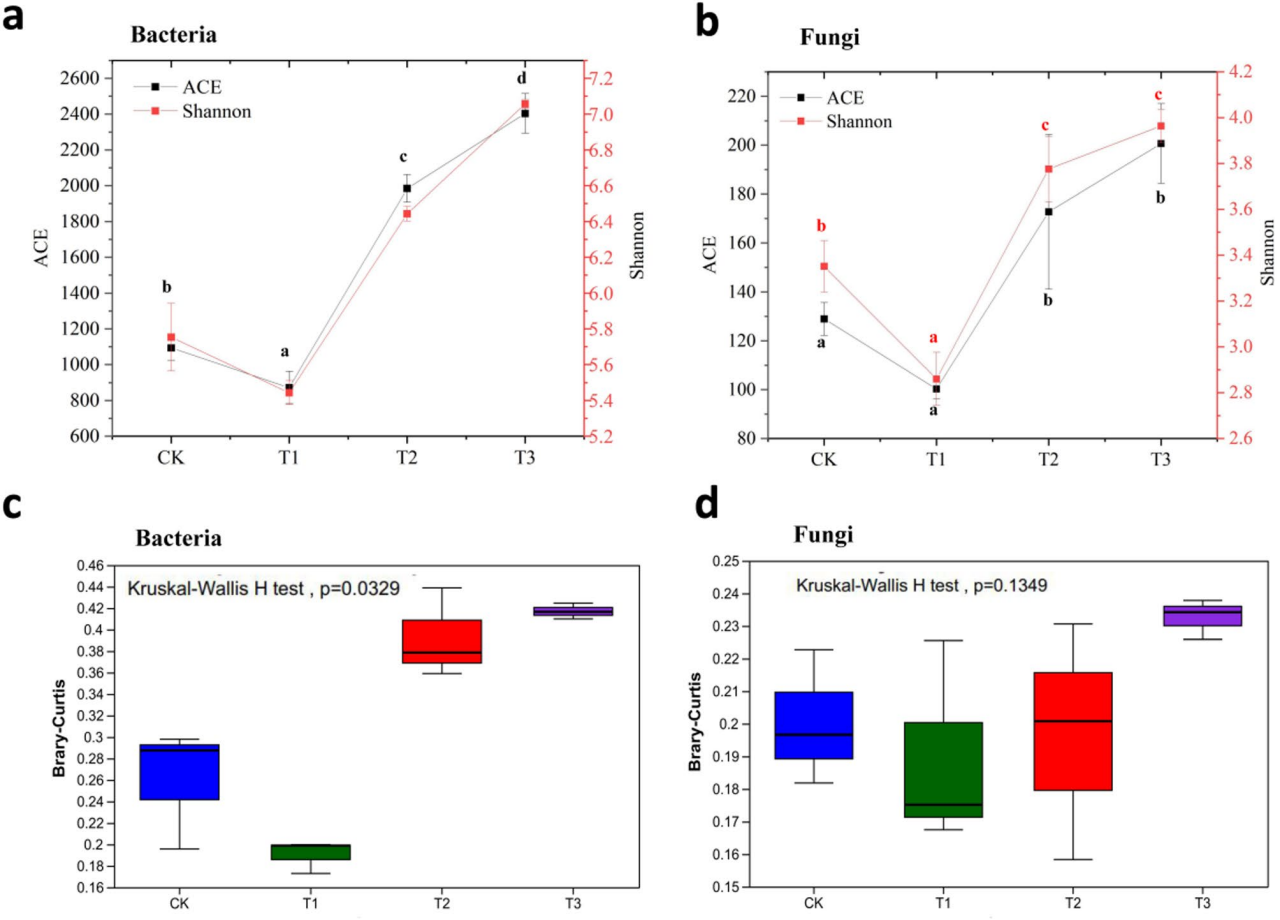
The analysis microbial diversity. (**a**) Alpha diversity of bacterial community based on ASV level; (**b**) Alpha diversity of fungal communities based on ASV level; (**c**) Box plots of analysis of similarities based on the Bray-Curtis dissimilarities of bacterial community; (**d**) Box plots of analysis of similarities based on the Bray-Curtis dissimilarities of bacterial community; Different lower-case letters refers to significant differences (*P* < 0.05) based on LSD method (The same as below)

A total of 583,599 bacterial reads were clustered into 20,552 ASVs, while 344,737 fungal reads were clustered into 1,801 ASVs. The distribution of bacterial reads varied insignificantly among the treatments, whereas fungal reads notably decreased in T1. The numbers of ASVs for both bacteria and fungi showed a slight decrease in T1, followed by a significant increase in T2, ultimately reaching the highest levels in soils treated with oyster shell powder for three years (Fig. S3). Alpha diversity analysis further demonstrated the impact of oyster shell powder application on microbial diversity. The ACE, which estimates species richness, and the Shannon index, a metric integrating both species richness and evenness (relative abundance distribution), were used to quantify biodiversity [30].‌ Soils amended with oyster shell powder exhibited a significantly higher level of biodiversity compared to the control, as indicated by the ACE and Shannon indices (Fig. 2a and b). Notably, microbial richness and diversity exhibited little variance or significant decreases in soils from T1, which aligned with the ASV distribution results. The structure of microbial communities was assessed using PCoA and Bray-Curtis dissimilarity matrices (Fig. S2, Fig. 2c and d). Soil bacterial and fungal community compositions clustered into four distinct groups (Fig. S2). Analysis of similarity indicated a significant difference in bacterial community composition (*p* = 0.0329), while no significant differences were observed among the fungal communities (*p* = 0.1349). This suggested that the application of oyster shell powder significantly affected bacterial structure and diversity, whereas the fungal community demonstrated greater stability in response to the application of oyster shell powder.

### Bacterial and fungal community composition

**Fig. 3:**
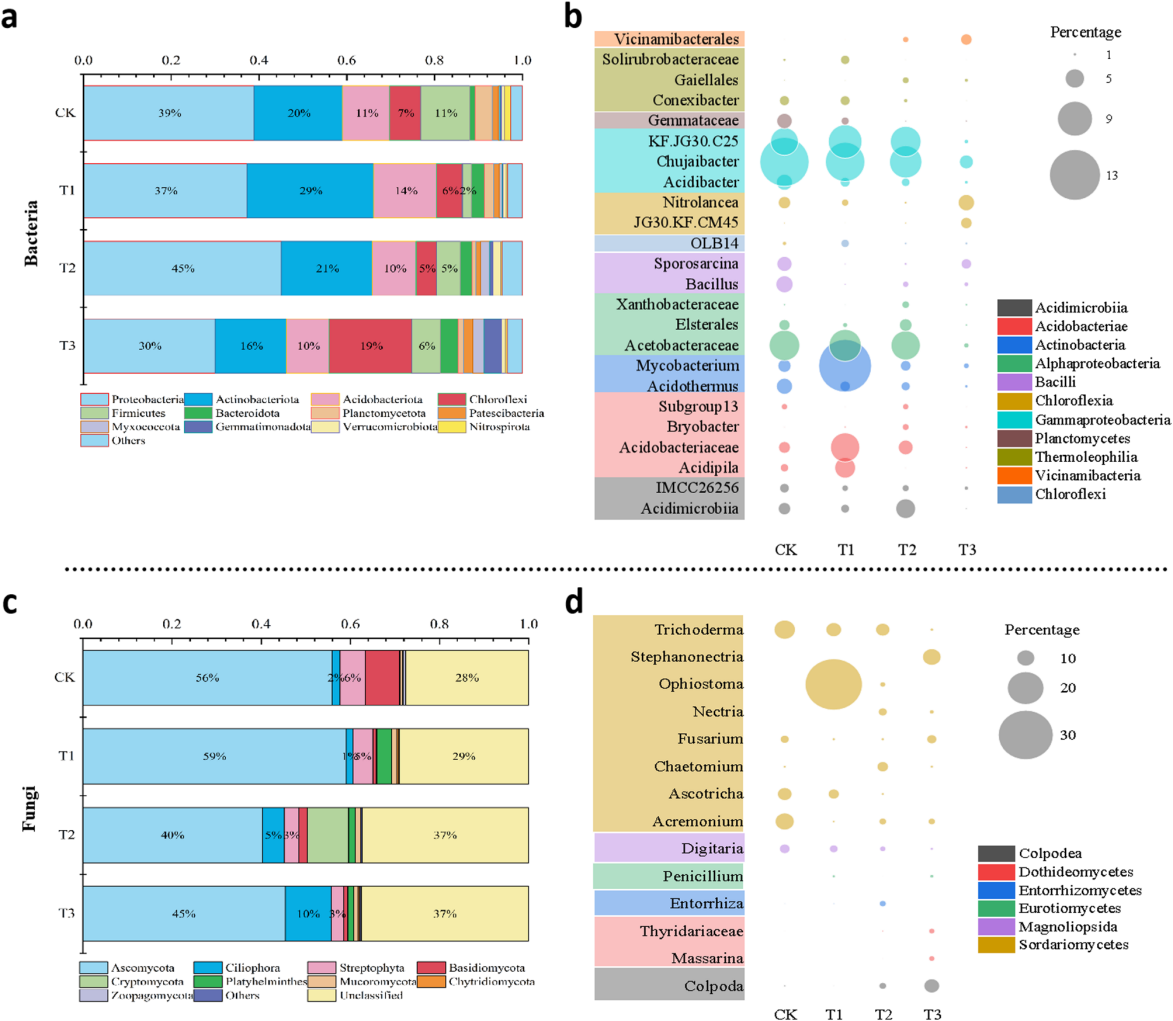
Taxonomic profiling of the microbial community composition. Bacterial community profiling at the phylum level (**a**) and at the genus level (**b**); Fungal community profiling at the phylum level (**c**) and at the genus level (**d**); Dot size in the panel c and d is proportional to the relative abundance of microbial groups. The genera shadowed in colorful boxes are from the same class group

Taxonomic analysis of 16 S rRNA gene revealed that Proteobacteria (38%), Actinobacteriota (21%), Acidobacteriota (12%), Chloroflexi (9%) and Firmicute (7%) were the most relatively abundant phyla across soils subjected to varying intensities of oyster shell powder application. Significant differences were observed among these phyla, with the exception of Acidobacteriota. Notably, soils treated with oyster shell powder for three years exhibited a significantly lower relative abundance of Actinobacteriota and Firmicutes when compared to the control. In contrast, Chloroflexi was significantly more abundant in soils amended with oyster shell powder compared to the control (Fig. 3a). Further, results indicated that 50 genera exhibited significant variations in relative abundance across different treatment groups, with those representing more than 1% relative abundance illustrated in Fig. 3b (Table S3-S4). These genera were categorized into 11 groups based on their class. The class Gammaproteobacteria included genera such as *Acidibacter*,* Chujaibacter* and *KF.JG30.C25*, which together accounted for 33% of the bacterial genera and were more prevalent in soils from the control and the first two years of oyster shell application. However, their relative abundance significantly decreased in soils after three years of application. *Mycobacterium*, belonging to the class Actinobacteria, along with *Acidobacteriaceae* and *Acidipila* from the class Acidobacteria, significantly increased in soils after one year of oyster shell application. Additionally, soils subjected to two years of oyster shell powder application significantly accumulated the genera *Gaiellales*,* Xanthobacteraceae*,* Bryobacter*, and *Acidimicrobiia*, while soils treated for three years significantly accumulated the genera *Vicinamibacterales*,* Nitrolancea*, and *JG30.KF.CM45.*

The taxonomic analysis of 18 S rRNA gene revealed that Ascomycota comprised 40 ~ 59% of the fungal phyla identified across the 12 soil samples, with its relative abundance significantly decreasing (*P* < 0.05) as the duration of oyster shell application increased (Fig. 3c). Streptophyta and Basidiomycota constituted for 3 ~ 6% of the total fungal community, and their relative abundances also decreased significantly in soils subjected to prolonged oyster shell application. In contrast, Ciliophora represented 4% of the fungal phyla and significantly increased its relative abundance in soils with two and three years of oyster shell application (Fig. 3c). At the genus level, a total of 31 genera exhibited significant variations in relative abundance across the different treatment groups, with those representing more than 10% relative abundance displayed in Fig. 3d (Table S5-S6). Most of these genera belonged to the class Sordariomycetes. Notably, *Trichoderma*,* Ascotricha* and *Acremonium* were more prevalent in soils without oyster shell application, while *Ophiostoma* showed a significant increase in relative abundance in soils with one year of oyster shell application. Additionally, soils treated with oyster shell powder for two years significantly accumulated the genera of *Nectria*,* Chaetomium*, and *Entorrhiza*. Soils with three years of oyster shell application demonstrated a significant increase in the genera *Stephanonectria*,* Thyridariaceae*, *Massarina* and *Colpoda*. Overall, the application of oyster shell powder significantly altered both bacterial and fungal communities in the acidified pomelo gardens. It is noteworthy that there remains limited understanding of the fungal community, as 32% of the fungi could not be identified.

### Effects of soil properties on the composition and diversity of microbial communities

**Fig. 4:**
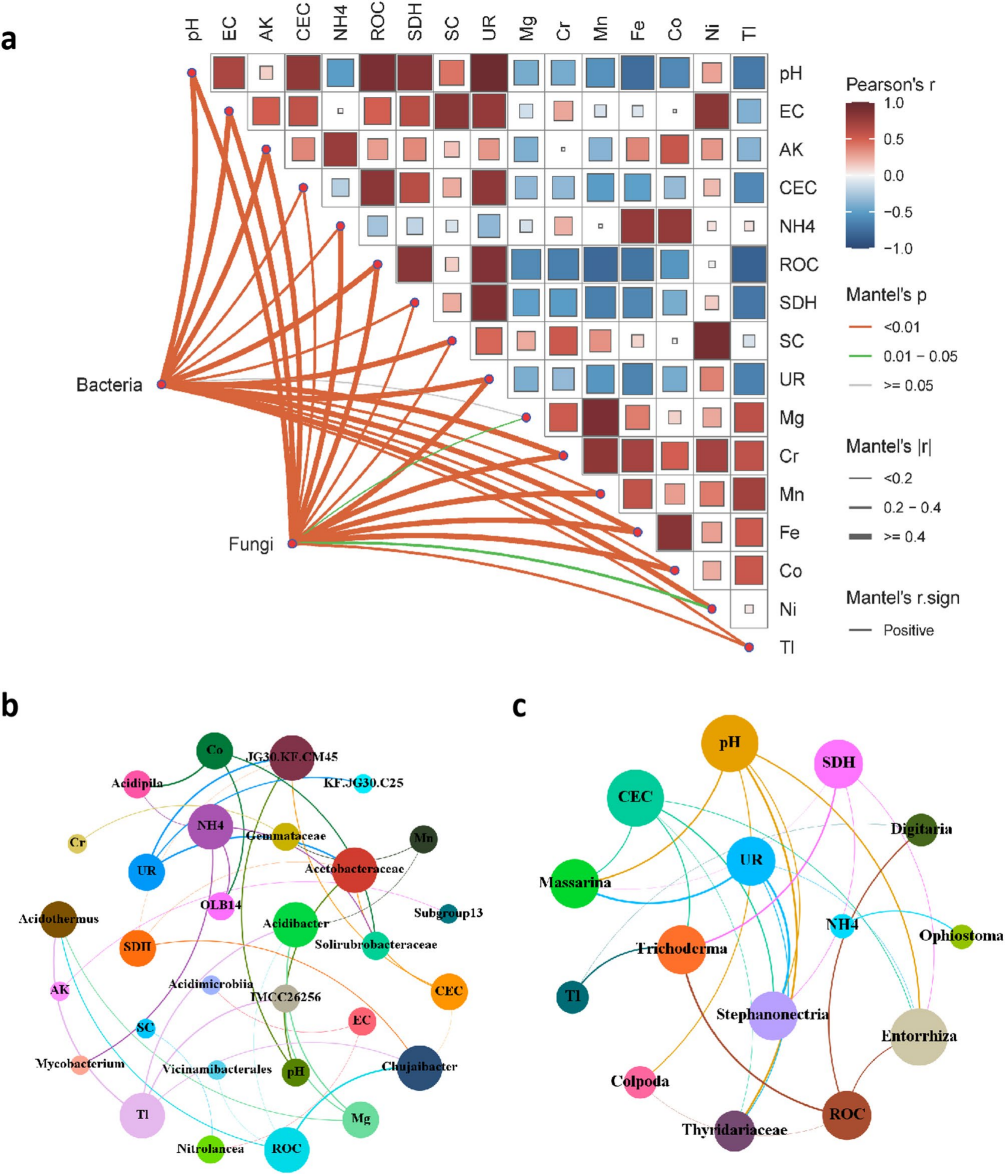
Correlation analysis of Soil properties and diversity (**a**), bacterial community (**b**) and fungal community (**c**) from the pomelo tree planting soils applied with oyster shell. (**a**): pairwise comparisons between environmental factors were shown in a color gradient, with individual boxes indicating the values of Pearson’s correlation coefficient. Beta diversity of bacterial and fungal communities (Bray-Curtis dissimilarity between samples) were related to each soil property by partial Mantel tests. The colored squares indicated the direction and strength of Pearson’s correlations; the size of each square reflects the absolute value of r. (**b**) and (**c**): edge widths between soil properties and microbial diversity indices scale with the magnitude of correlation coefficients (|r|), while edge colors categorize *p*-value ranges; the size of the circle represented the number of related lines; the thickness of the lines indicated the size of the correlation

Correlations between the diversity and composition of microbial communities and soil properties were identified (Fig. 4; Table S7-S10). Bacterial diversity exhibited significant positive correlations with most soil properties, with the exception of Mg. In contrast, fungal diversity was significantly positively correlated with all detected soil properties (Fig. 4a; Table S9). Additionally, soil pH was significantly positively correlated with EC, CEC, ROC and SDH, but negatively correlated with Fe. Soil EC showed significant positive correlations with pH, CEC, SDH, SC and UR, while soil CEC was significantly positively correlated with pH, EC, SDH and UR. Soil ROC exhibited significant positive correlations with pH, CEC, SDH and UR, but significant negative correlations with the metals Mg, Cr, Mn and Fe. Furthermore, soil SDH was significantly positively correlated with pH, EC, CEC and ROC, while negatively correlated with UR and the metals Mn and Fe. Additionally, the metal Tl was significantly negatively correlated with pH, CEC, ROC, SDH and UR, but positively correlated with the metals Mg, Cr and Mn (Fig. 4a; Table S10).

Correlations between genera of bacterial and fungal communities and soil properties were also assessed (Fig. 4b and c; Table S7-S8). Specifically, *Acetobacteraceae* and *JG30.KF.CM45* showed significant correlations with pH, CEC, SDH and UR, although the directions of these correlation were opposite. *Acidothermus* and *Acidibacter* exhibited significant correlations with ROC, Mg and Tl. The genera *Chujaibacter* also demonstrated significant correlations with CEC, ROC, Tl and SDH. Additionally, the topological features of the network revealed that Tl, NH_4_^+^ and ROC had the most significant correlations with the distribution of the bacterial community (Fig. 4a). Similarly, the fungal genera *Massarina*,* Enthrrhiza* and *Stephanonectria* showed significantly positive correlations with pH, CEC, SDH and UR, while *Trichoderma* exhibited significant correlations with CEC, ROC, Tl and SDH. Overall, environmental factors such as pH and CEC displayed the most significant correlations with the fungal community (Fig. 4b).

### Correlation analysis of microbial groups

The microbial correlation under oyster shell amendment was conducted using Pearson’s analysis (Table S11; Table S12). Strong positive correlations emerged between acidophilic genera like *Acidothermus* and *Acidibacter*, both suppressed by pH elevation, as well as *OLB14* and *Solirubrobacteraceae*. Key negative correlations included *Vicinamibacterales* with *Conexibacter*, and *JG30.KF.CM45* with *Acetobacteraceae.* Notably, the pathogenic genus *Mycobacterium* showed strong positive associations with acid-tolerant *Acidipila* and *OLB14*, underscoring their shared resilience to acidic stress. In fungal community, *Digitaria* displayed positive associations with *Ascotricha* and *Trichoderma.* Key negative correlations emerged between *Digitaria* and multiple taxa, including *Colpoda*, *Thyridariaceae* and *Entorrhiza*, approaching statistical significance. The strongest antagonistic relationship was observed between *Trichoderma* and *Entorrhiza.* Interestingly, *Digitaria* displayed positive associations with *Ascotricha* and *Trichoderma*, forming a distinct cluster.

### Functional profile prediction

**Fig. 5:**
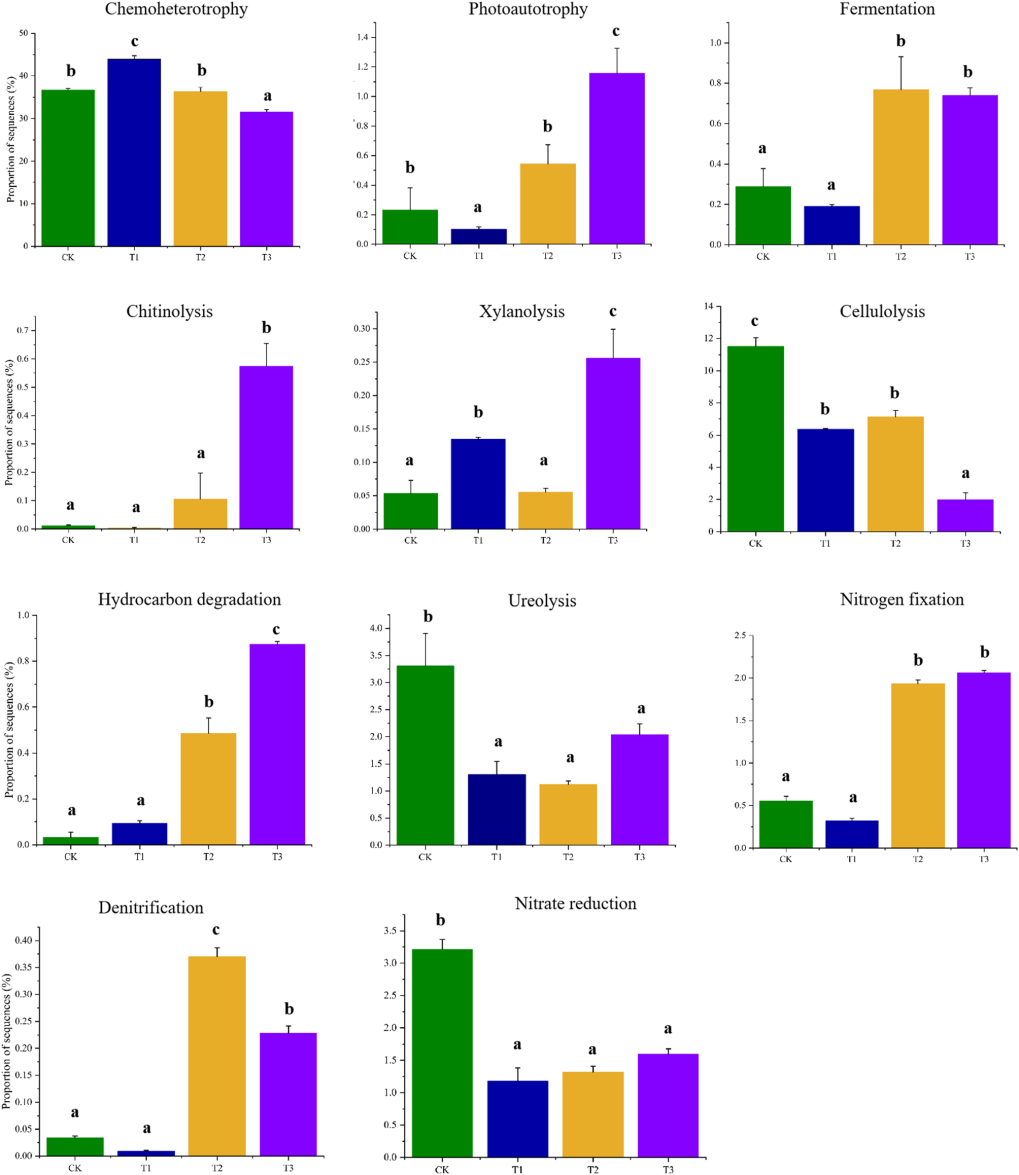
Bar-graph showing the differential relative abundance of soil bacterial functional groups from soils applied with different years of oyster shell. The prediction was made using the FAPROTAX database

**Fig. 6:**
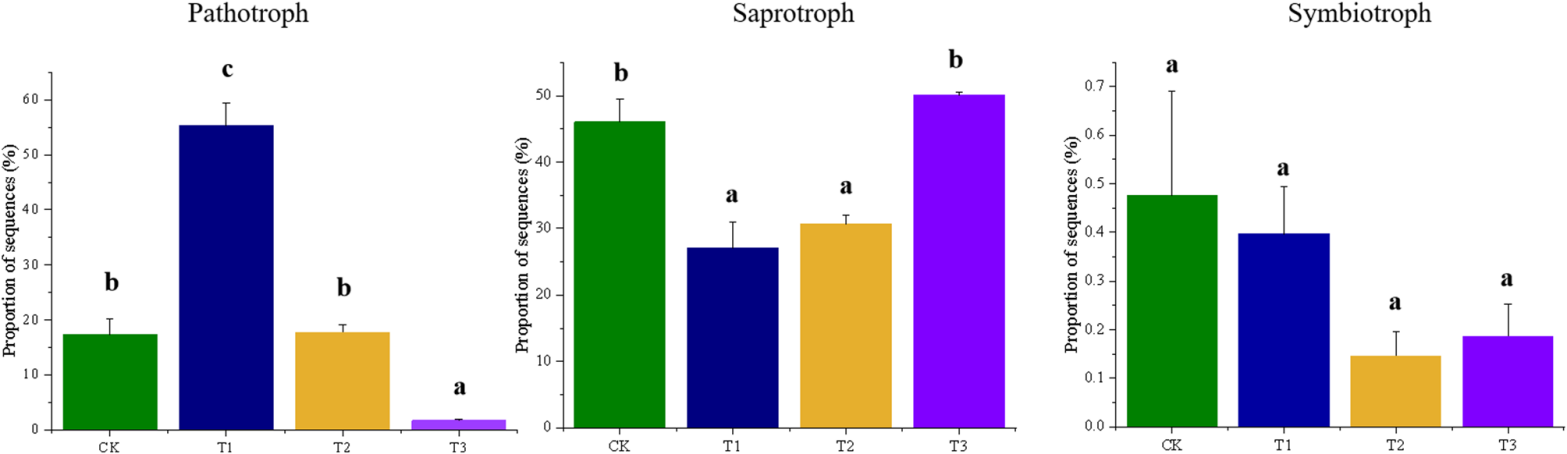
Bar-graph showing the differential relative abundance of soil fungal functional groups from soils applied with different years of oyster shell. The prediction was made using the FUNGuild database

The functional groups within both bacterial and fungal communities were predicted and displayed in Figs. 5 and 6. Three years of oyster shell application significantly altered soil microbial communities. Chemoheterotrophic bacteria decreased while photoautotrophic bacteria increased. Fermentative, chitinolytic, xylanolytic, and hydrocarbon-degrading bacteria became more abundant, whereas cellulolytic bacteria declined. Ureolytic and nitrate-reducing bacteria decreased, while nitrogen-fixing and denitrifying bacteria increased after two to three years of treatment. In fungal communities, pathogenic, saprophytic, and symbiotic types dominated (> 60% of sequences), with saprophytes exceeding 40%. Saprophytic fungi initially declined in the first two years but recovered to baseline by the third year. Pathogenic fungi, dominant in pre-treatment acidified soils, significantly decreased after three years of oyster shell application. Symbiotic fungi remained a minor component with no significant changes across treatments.

## Discussion

### Soil property modifications and pH-driven heavy metal mitigation

Our results indicated that three-year application of oyster shell powder significantly enhanced soil health and microbial activity in acidified pomelo orchards. While oyster shells’ alkaline properties effectively neutralize soil acidity and improve fruit quality long-term [5–7], their comprehensive effects on soil properties and micro-ecology require further investigation. In this study, the measured soil properties collectively reflect critical aspects of soil health [31]. ‌pH‌ governs soil acidity/alkalinity, modulating nutrient availability, while EC indicates salinity levels that may synergistically exacerbate metal toxicity to plants. CEC‌ and SOC‌ define the soil’s capacity to retain cationic nutrients, with SOC further acting as a metal-chelating agent to mitigate bioavailability. ‌AK‌ and ‌NH_4_⁺-N‌ represent plant-accessible macronutrients essential for growth. Enzymatic activities—SDH, UR and SC—serve as sensitive bioindicators of soil microbial functions and nutrient turnover. Finally, ‌total metal content‌ reflects geological and anthropogenic inputs. Collectively, these parameters provide a holistic framework to assess soil functionality, pollution resilience, and ecosystem recovery potential [32]. In this study, strong correlations were observed between soil pH and EC, CEC and ROC, with the highest values of these properties recorded in the T3 treatment (three years of oyster shell application) compared to other experimental groups. The significant increase in CEC under oyster shell treatment aligns with its calcium-rich composition, which promotes soil aggregation and organic-mineral complexation [2]. Concurrently, higher ROC suggests enhanced microbial activity and organic nutrient mineralization, facilitating nutrient supply. EC and the content of AK in the soil also exhibited a marked increase, demonstrating enhanced potassium bioavailability. These collectively validate that oyster shell-based conditioners can enhance the soil’s capability to retain and supply nutrients [9, 33, 34]. Furthermore, we observed a significant reduction in the contents of heavy metals, specifically Cr and Tl, following the application of oyster shell powder, with concentrations reaching their lowest levels after three years. In this study, soils in Pinghe County, Fujian Province, ‌have a historical background of heavy metal contamination‌, consistent with long-term intensive agricultural practices. The ‌control‌ data (Table 1) clearly indicated elevated heavy metal levels compared to common agricultural soil thresholds. For example, the content of Cr‌ was approaching the maxmium content of Cr in normal farmland soil (20 mg.kg^− 1^) [35], while the content of Tl was‌ significantly above the natural abundance (0.3 mg.kg^− 1^) [36]. While the positive effect of oyster shell on heavy metal-contaminated soils, particularly those contaminated with cadmium (Cd) and Pb, has been well-documented [37, 38], our study provides the first evidence of its efficacy in mitigating Cr and Tl contamination in pomelo-planting soils. However, the results also indicated a potential risk of Mg loss, a factor that warrants further investigation.

### pH-mediated microbial community restructuring and functional shifts

Soil microbial characteristics, which serve as bio-indicators of soil health, typically respond swiftly but variably to changes in soil properties and fertilization practices. For instance, Zheng et al. [6] observed that oyster shell application reduced the diversity of inter-root soil bacteria in tomato plants, whereas Shen et al. [39] reported that oyster shell application increased bacterial diversity in tobacco-planting soils. In our study, we observed that the richness and diversity of microbial community were actively influenced by various soil properties (Fig. 4) and significantly increased with the application of oyster shell (Fig. 2). Additionally, the bacterial community composition exhibited significant variations across the sampled soils during the oyster shell application (Fig. 2), suggesting that bacterial communities were more responsive to oyster shell amendments while fungal communities remained relatively stable throughout the application period. The observed mismatch between phylum/genus-level fungal shifts and the non-significant beta diversity index likely reflects localized responses of specific taxa to environmental pressures (e.g., oyster shell amendments), while broader community resilience persists via functional redundancy or compensatory interactions. Additionally, finer taxonomic-resolution differences may arise disproportionately from fluctuations in keystone taxa, which strongly affect narrow-scale diversity metrics but exert minimal influence on overall beta diversity patterns. These findings aligned with those of Choma et al. [40] who also reported that bacteria, but not fungi, responded rapidly and consistently to soil acidification in forest ecosystem. Interestingly, The T1 sample showed the lowest microbial diversity, likely due to heavy rainfall and prolonged fertilizer use in 2021, which worsened soil acidification and potentially counteracted the oyster shell amendment’s benefits, as evidenced by its lower pH.

Proteobacteria, Actinobacteria and Acidobacteria dominated southern pomelo orchard soils, aligning with findings from other citrus-growing regions [41, 42]. The application of oyster shell significantly increased the relative abundances of Chloroflexi, Bacteroidota, and Gemmatimonadota, which aligns closely with the findings of Chen et al. [42]. Chloroflexi is known for its rich metabolic pathways and its significant role in the biogeochemical cycles of carbon and nitrogen [43, 44]. Specifically, the genus *Nitrolancea* (Chloroflexi) is a chemolithoautotrophic, nitrite-oxidizing bacterium that can use sunlight as an energy source. The increased relative abundance of *Nitrolancea* observed in soils with three years of oyster shell application not only contributes to the overall soil health [45] but also supports the high proportions of photoautotrophic groups, as shown in Fig. 5. *Vicinamibacterales* (Acidobacteriota) showed high abundance in oyster shell-amended soils, consistent with findings from rice-frog [46] and pea-coal systems [47], where it indicates healthy agroecosystems and improves phosphorus utilization [48]. In contrast, prolonged oyster shell application reduced *Acidibacter* and *Chujaibacter* (Proteobacteria) abundance (Fig. 3), genera positively correlated with Tl, Mn and Mg (Fig. 4) and known for heavy metal degradation in contaminated soils [49, 50]. Additionally, *Acidibacter* and *Chujaibacter* have also been associated with soil-borne diseases [51–54]. Similarly, oyster shell application reduced *Mycobacterium* (abundant in T1 soils) and *Acidothermus* (Actinobacteria) (Fig. 3) — genera associated with waterlogged/diseased soils [53] and acidic/degraded environments [54], respectively, with *Acidothermus* correlating with Tl. Their decline suggests improved soil conditions, though pathogenic roles require verification. Additionally, corrlation analysis demonstrated the strong positive correlations among acidophiles (*Acidothermus*,* Acidibacter*) and their negative relationships with alkaliphiles (*Nitrolancea*, *Chloroflexi*) reveal a pH-driven succession where neutralization favors alkaline-tolerant groups (Table S11).

Predicting the functional group response to oyster shell application is crucial for evaluating its potential effects on soil ecosystems. Functional analysis indicated that most bacteria in the soils relied on organic matter degradation as their primary carbon and energy source. The application of oyster shell significantly promoted processes such as chitinolysis, photoautotrophy, fermentation, hydrocarbon degradation, xylanolysis, nitrogen fixation, and denitrification. However, it also led to a decrease in nitrate reduction. The decline in nitrate reduction, coupled with enhanced nitrogen fixation and denitrification, may highlight a trade-off in nitrogen cycling dynamics under oyster shell application. Additionally, the introduction of large amounts of chitosan into the soil may contribute to the formation of soil granular structure and enhance plant resistance to stress, as noted by Song et al. [55]. Notably, despite inhibited ureolytic bacterial groups in functional predictions (Fig. 5), urease activity increased in soils treated with oyster shell (Table 1). Similarly, bacterial groups involved in cellulolysis were also inhibited, which contrasts with the findings of Wang et al. [56] and Zhang et al. [57], who proposed that cellulolysis tends to be enhanced during soil remediation. The observed disparity could arise from fungal contributions via undercharacterized ureolytic pathways overlooked by bacterial-centric functional pipelines [58], and oyster shell-induced physicochemical shifts (e.g., pH/Ca²⁺ elevation) stabilizing extracellular urease or enhancing substrate accessibility [59, 60]. Furthermore, these findings underscore the complexity of the interaction between oyster shell amendments and the soil microbial community, suggesting the need for further verification.

Fungi also play a critical role in soil health. Ascomycota was the predominant fungal phylum across the soils, but its relative abundance significantly decreased, particularly in the genera *Trichoderma* and *Acremonium*, following oyster shell application (Fig. 3c and d). The distribution of *Trichoderma* showed significant correlations with Tl, CEC, and ROC (Fig. 4), consistent with its known role as a plant growth-promoting fungus [61, 62]. Similarly, *Acremonium* produces bioactive metabolites and suppresses nematodes [63, 64]. These results suggested that oyster shell application may have altered fungal-plant interactions through soil property changes. The reduced *Trichoderma* abundance indicated improved soil health with lower Tl levels, aligning with Sun et al. [65] who reported its dominance in Tl-contaminated soils. Oyster shell-treated soils showed elevated Ophiostoma (Sordariomycetes) abundance in the first year (Fig. 3d), a genus containing human pathogens and plant pests [66, 67], potentially explaining the high pathogenic group proportion in Tl-contaminated soils (Fig. 6). On the other side, oyster shell application promoted the accumulation of genera such as *Stephanonectria* and *Colpoda* (Fig. 3d), both of which are typically enriched in soils supporting healthy plants, as shown by studies in wheat planting soils [68] and leguminous plant soils [69]. Furthermore, *Colpoda* has been suggested as a bioremediator for Cd contamination [70] and a carrier for plant-beneficial bacteria in soils [63]. Both genera’s distributions strongly correlated with soil pH (Fig. 4c), demonstrating that oyster shells simultaneously remediate Cd through microbial enrichment and neutralize acidity. This pH-mediated shift likely restructures microbial communities, favoring plant-beneficial organisms - consistent with known *Colpoda*-mediated bacterial recruitment in rhizospheres [69, 70].

## Conclusion

Our study reveals that three-year oyster shell powder application enhances soil carbon cycling and reduces heavy metal pollution, while significantly increasing microbial diversity. The treatment promoted beneficial microorganisms involved in carbohydrate degradation and reduced pathogenic taxa, with specific microbial groups showing strong correlations with Tl/Cd detoxification. While bacterial communities responded markedly to treatment, fungal community exhibited fewer sensitive responses to oyster shell application, showing reduced plant-interaction and cellulose-decomposition functions. These findings demonstrate oyster shell’s potential for improving acidic soil ecosystems. Future research should combine metagenomics, nanoscale analyses (SEM-EDS/XPS), and meta-transcriptomics to better understand microbe-shell interactions and active microbial functions. Additionally, while read-length filtering ensured data quality, it limited rare taxon detection. We will adopt ‌deeper sequencing‌ and ‌long-read technologies‌ in follow-up studies to better capture microbial diversity.

## Electronic supplementary material

Below is the link to the electronic supplementary material.

Supplementary Material 1

## Data Availability

No datasets were generated or analysed during the current study.
