# Supplementary material for: Effect of applying oyster shell powder on soil properties and microbial diversity in the acidified soils of pomelo garden
Source: Environ Microbiome. 2025 May 24;20:57. doi: 10.1186/s40793-025-00721-6 (PMC12103764; doi:10.1186/s40793-025-00721-6)
Supplement: Supplementary file 1 — Supplementary Material 1 [file 40793_2025_721_MOESM1_ESM.docx]

**Supplementary Material**

**Effect of applying oyster shell powder on soil properties and microbial diversity in the acidified soils of pomelo garden**

Yuanyuan Li^1^, Jing Yang^1^, Jingjing Wei^1^, Qiong Zhang^1^, Lixia Zhu^1^, Xiaohuang Chen^2,3*^

**Supplementary Tables**

Table S1 The basic sequence information statistics

|  | **Sample** | **Raw sequence** | **Base(bp)** | **Mean length(bp)** | **Min length(bp)** | **Max length(bp)** | **Qualified sequence** |
| --- | --- | --- | --- | --- | --- | --- | --- |
| **Bacteria** | CK_1 | 65742 | 27442039 | 417 | 235 | 472 | 42779 |
|  | CK_2 | 201282 | 84031174 | 417 | 233 | 525 | 141319 |
|  | CK_3 | 70966 | 29773990 | 420 | 294 | 471 | 41090 |
|  | T1_1 | 70491 | 29396060 | 417 | 229 | 483 | 46196 |
|  | T1_2 | 59620 | 24892559 | 418 | 202 | 465 | 39802 |
|  | T1_3 | 60005 | 24985209 | 416 | 314 | 475 | 39385 |
|  | T2_1 | 82529 | 34463060 | 418 | 245 | 467 | 43952 |
|  | T2_2 | 63716 | 26567151 | 417 | 337 | 454 | 37665 |
|  | T2_3 | 62796 | 26181231 | 417 | 236 | 519 | 37059 |
|  | T3_1 | 64769 | 26978659 | 417 | 291 | 465 | 38736 |
|  | T3_2 | 59258 | 24674678 | 416 | 234 | 467 | 37665 |
|  | T3_3 | 64267 | 26762639 | 416 | 297 | 525 | 37951 |
| **Fungi** | CK_1 | 61694 | 23536923 | 382 | 256 | 414 | 27609 |
|  | CK_2 | 65587 | 25028065 | 382 | 257 | 407 | 30556 |
|  | CK_3 | 61579 | 23494405 | 382 | 257 | 404 | 29600 |
|  | T1_1 | 48401 | 18458688 | 381 | 368 | 387 | 24147 |
|  | T1_2 | 45141 | 17213202 | 381 | 258 | 402 | 23036 |
|  | T1_3 | 46451 | 17711766 | 381 | 371 | 406 | 23047 |
|  | T2_1 | 77707 | 29604096 | 381 | 259 | 419 | 36371 |
|  | T2_2 | 58050 | 22136612 | 381 | 367 | 405 | 28844 |
|  | T2_3 | 67534 | 25719073 | 381 | 260 | 433 | 33846 |
|  | T3_1 | 60563 | 23028319 | 380 | 257 | 423 | 27847 |
|  | T3_2 | 61551 | 23427455 | 381 | 299 | 433 | 30571 |
|  | T3_3 | 61782 | 23513249 | 381 | 259 | 415 | 29264 |

**Table S2** The primary data of alpha diversity indices.

|  | **Sample** | **ACE** | **Chao** | **Shannon** | **Simpson** | **Coverage** |
| --- | --- | --- | --- | --- | --- | --- |
| **Bacteria** | CK_1 | 1044.61 | 1038.92 | 5.71 | 0.01 | 1.00 |
|  | CK_2 | 2246.46 | 2293.33 | 5.96 | 0.01 | 0.99 |
|  | CK_3 | 1141.59 | 1133.38 | 5.59 | 0.01 | 1.00 |
|  | T1_1 | 974.67 | 964.05 | 5.52 | 0.01 | 1.00 |
|  | T1_2 | 840.26 | 836.75 | 5.40 | 0.01 | 1.00 |
|  | T1_3 | 801.71 | 799.41 | 5.41 | 0.01 | 1.00 |
|  | T2_1 | 2456.94 | 2410.50 | 6.41 | 0.01 | 1.00 |
|  | T2_2 | 2038.91 | 2034.26 | 6.43 | 0.01 | 1.00 |
|  | T2_3 | 1930.00 | 1930.00 | 6.49 | 0.01 | 1.00 |
|  | T3_1 | 2453.07 | 2440.32 | 7.06 | 0.00 | 1.00 |
|  | T3_2 | 2277.53 | 2273.39 | 7.03 | 0.00 | 1.00 |
|  | T3_3 | 2481.15 | 2473.70 | 7.08 | 0.00 | 1.00 |
| **Fungi** | CK_1 | 129.24 | 129.00 | 3.44 | 0.05 | 1.00 |
|  | CK_2 | 135.54 | 135.14 | 3.39 | 0.06 | 1.00 |
|  | CK_3 | 122.00 | 122.00 | 3.22 | 0.07 | 1.00 |
|  | T1_1 | 105.00 | 105.00 | 2.81 | 0.14 | 1.00 |
|  | T1_2 | 97.00 | 97.00 | 2.99 | 0.11 | 1.00 |
|  | T1_3 | 99.00 | 99.00 | 2.78 | 0.15 | 1.00 |
|  | T2_1 | 205.30 | 203.65 | 3.89 | 0.04 | 1.00 |
|  | T2_2 | 142.14 | 142.00 | 3.62 | 0.05 | 1.00 |
|  | T2_3 | 170.80 | 169.91 | 3.82 | 0.04 | 1.00 |
|  | T3_1 | 187.22 | 187.00 | 3.89 | 0.04 | 1.00 |
|  | T3_2 | 195.91 | 196.14 | 3.97 | 0.03 | 1.00 |
|  | T3_3 | 218.83 | 218.33 | 4.03 | 0.03 | 1.00 |

**Table S3** ANOVA analysis of bacterial community on the genus level.

|  | | **Square Sum** | **Variance** | **Mean square** | **F** | **Significance** |
| --- | --- | --- | --- | --- | --- | --- |
| ***Acidipila*** | **Within groups** | .003 | 3 | .001 | 18.877 | .001 |
|  | **Between groups** | .000 | 8 | .000 |  |  |
|  | **Total** | .004 | 11 |  |  |  |
| ***Acidobacteriaceae*** | **Within groups** | .000 | 3 | .000 | 19.578 | .000 |
|  | **Between groups** | .000 | 8 | .000 |  |  |
|  | **Total** | .000 | 11 |  |  |  |
| ***Bryobacter*** | **Within groups** | .004 | 3 | .001 | 42.819 | .001 |
|  | **Between groups** | .000 | 5 | .000 |  |  |
|  | **Total** | .004 | 8 |  |  |  |
| ***Granulicella*** | **Within groups** | .000 | 3 | .000 | 388.438 | .000 |
|  | **Between groups** | .000 | 6 | .000 |  |  |
|  | **Total** | .000 | 9 |  |  |  |
| ***Subgroup13*** | **Within groups** | .000 | 3 | .000 | 5.172 | .034 |
|  | **Between groups** | .000 | 7 | .000 |  |  |
|  | **Total** | .000 | 10 |  |  |  |
| ***Acidothermus*** | **Within groups** | .000 | 3 | .000 | 14.866 | .002 |
|  | **Between groups** | .000 | 7 | .000 |  |  |
|  | **Total** | .000 | 10 |  |  |  |
| ***Mycobacterium*** | **Within groups** | .000 | 3 | .000 | 21.282 | .001 |
|  | **Between groups** | .000 | 6 | .000 |  |  |
|  | **Total** | .000 | 9 |  |  |  |
| ***Streptomyces*** | **Within groups** | .001 | 3 | .000 | 32.929 | .000 |
|  | **Between groups** | .000 | 7 | .000 |  |  |
|  | **Total** | .001 | 10 |  |  |  |
| ***Acetobacteraceae*** | **Within groups** | .027 | 3 | .009 | 598.489 | .000 |
|  | **Between groups** | .000 | 8 | .000 |  |  |
|  | **Total** | .027 | 11 |  |  |  |
| ***Elsterales*** | **Within groups** | .000 | 3 | .000 | 386.300 | .000 |
|  | **Between groups** | .000 | 7 | .000 |  |  |
|  | **Total** | .000 | 10 |  |  |  |
| ***Micropepsaceae*** | **Within groups** | .009 | 3 | .003 | 97.389 | .000 |
|  | **Between groups** | .000 | 7 | .000 |  |  |
|  | **Total** | .010 | 10 |  |  |  |
| ***Sphingomonas*** | **Within groups** | .001 | 3 | .000 | 63.998 | .000 |
|  | **Between groups** | .000 | 7 | .000 |  |  |
|  | **Total** | .001 | 10 |  |  |  |
| ***Xanthobacteraceae*** | **Within groups** | .000 | 3 | .000 | 62.763 | .000 |
|  | **Between groups** | .000 | 6 | .000 |  |  |
|  | **Total** | .000 | 9 |  |  |  |
| ***Chujaibacter*** | **Within groups** | .002 | 3 | .001 | 22.278 | .003 |
|  | **Between groups** | .000 | 5 | .000 |  |  |
|  | **Total** | .002 | 8 |  |  |  |
| ***KF.JG30.C25*** | **Within groups** | .002 | 3 | .001 | 134.972 | .000 |
|  | **Between groups** | .000 | 8 | .000 |  |  |
|  | **Total** | .002 | 11 |  |  |  |
| ***Nitrospira*** | **Within groups** | .013 | 3 | .004 | 56.921 | .000 |
|  | **Between groups** | .001 | 8 | .000 |  |  |
|  | **Total** | .013 | 11 |  |  |  |
| ***Gemmataceae*** | **Within groups** | .010 | 3 | .003 | 36.312 | .000 |
|  | **Between groups** | .001 | 8 | .000 |  |  |
|  | **Total** | .011 | 11 |  |  |  |
| ***Haliangium*** | **Within groups** | .000 | 3 | .000 | 35.754 | .000 |
|  | **Between groups** | .000 | 8 | .000 |  |  |
|  | **Total** | .000 | 11 |  |  |  |
| ***Sandaracinaceae*** | **Within groups** | .002 | 3 | .001 | 144.662 | .000 |
|  | **Between groups** | .000 | 5 | .000 |  |  |
|  | **Total** | .002 | 8 |  |  |  |
| ***Saccharimonadales*** | **Within groups** | .000 | 3 | .000 | 35.759 | .000 |
|  | **Between groups** | .000 | 6 | .000 |  |  |
|  | **Total** | .000 | 9 |  |  |  |
| ***Conexibacter*** | **Within groups** | .000 | 3 | .000 | 54.292 | .000 |
|  | **Between groups** | .000 | 7 | .000 |  |  |
|  | **Total** | .000 | 10 |  |  |  |
| ***Gaiellales*** | **Within groups** | .000 | 3 | .000 | 6.286 | .028 |
|  | **Between groups** | .000 | 6 | .000 |  |  |
|  | **Total** | .000 | 9 |  |  |  |
| ***Solirubrobacteraceae*** | **Within groups** | .001 | 3 | .000 | 97.577 | .000 |
|  | **Between groups** | .000 | 8 | .000 |  |  |
|  | **Total** | .001 | 11 |  |  |  |
| ***Solirubrobacterales*** | **Within groups** | .000 | 3 | .000 | 623.312 | .000 |
|  | **Between groups** | .000 | 7 | .000 |  |  |
|  | **Total** | .000 | 10 |  |  |  |
| ***Vicinamibacterales*** | **Within groups** | .001 | 3 | .000 | 72.685 | .000 |
|  | **Between groups** | .000 | 8 | .000 |  |  |
|  | **Total** | .001 | 11 |  |  |  |

**Table S4** Multiple comparisons analysis of bacterial community on the genus level through the LSD method.

|  | | **(I)** | **(J)** | **Mean value (I-J)** | **Standard error** | **Significance** | **95% Confidence interval** | |
| --- | --- | --- | --- | --- | --- | --- | --- | --- |
|  |  | **Group** | **Group** |  |  |  | **Upper limit** | **Lower limit** |
| ***Acidipila*** | **LSD** | **0** | **1** | 0.009 | 0.006 | 0.179 | -0.005 | 0.023 |
|  |  |  | **2** | -.018304146* | 0.006 | 0.017 | -0.032 | -0.004 |
|  |  |  | **3** | .026669185* | 0.006 | 0.002 | 0.013 | 0.041 |
|  |  | **1** | **0** | -0.009 | 0.006 | 0.179 | -0.023 | 0.005 |
|  |  |  | **2** | -.027289817* | 0.006 | 0.002 | -0.041 | -0.013 |
|  |  |  | **3** | .017683514* | 0.006 | 0.020 | 0.004 | 0.032 |
|  |  | **2** | **0** | .018304146* | 0.006 | 0.017 | 0.004 | 0.032 |
|  |  |  | **1** | .027289817* | 0.006 | 0.002 | 0.013 | 0.041 |
|  |  |  | **3** | .044973331* | 0.006 | 0.000 | 0.031 | 0.059 |
|  |  | **3** | **0** | -.026669185* | 0.006 | 0.002 | -0.041 | -0.013 |
|  |  |  | **1** | -.017683514* | 0.006 | 0.020 | -0.032 | -0.004 |
|  |  |  | **2** | -.044973331* | 0.006 | 0.000 | -0.059 | -0.031 |
| ***Acidobacteriaceae*** | **LSD** | **0** | **1** | .007690440* | 0.002 | 0.002 | 0.004 | 0.012 |
|  |  |  | **2** | .006728010* | 0.002 | 0.005 | 0.003 | 0.011 |
|  |  |  | **3** | .013465016* | 0.002 | 0.000 | 0.009 | 0.018 |
|  |  | **1** | **0** | -.007690440* | 0.002 | 0.002 | -0.012 | -0.004 |
|  |  |  | **2** | -0.001 | 0.002 | 0.600 | -0.005 | 0.003 |
|  |  |  | **3** | .005774576* | 0.002 | 0.011 | 0.002 | 0.010 |
|  |  | **2** | **0** | -.006728010* | 0.002 | 0.005 | -0.011 | -0.003 |
|  |  |  | **1** | 0.001 | 0.002 | 0.600 | -0.003 | 0.005 |
|  |  |  | **3** | .006737005* | 0.002 | 0.005 | 0.003 | 0.011 |
|  |  | **3** | **0** | -.013465016* | 0.002 | 0.000 | -0.018 | -0.009 |
|  |  |  | **1** | -.005774576* | 0.002 | 0.011 | -0.010 | -0.002 |
|  |  |  | **2** | -.006737005* | 0.002 | 0.005 | -0.011 | -0.003 |
| ***Bryobacter*** | **LSD** | **0** | **1** | -.031404877* | 0.005 | 0.002 | -0.045 | -0.018 |
|  |  |  | **2** | .018268167* | 0.006 | 0.022 | 0.004 | 0.033 |
|  |  |  | **3** | .015286435* | 0.006 | 0.041 | 0.001 | 0.030 |
|  |  | **1** | **0** | .031404877* | 0.005 | 0.002 | 0.018 | 0.045 |
|  |  |  | **2** | .049673044* | 0.005 | 0.000 | 0.037 | 0.063 |
|  |  |  | **3** | .046691312* | 0.005 | 0.000 | 0.034 | 0.060 |
|  |  | **2** | **0** | -.018268167* | 0.006 | 0.022 | -0.033 | -0.004 |
|  |  |  | **1** | -.049673044* | 0.005 | 0.000 | -0.063 | -0.037 |
|  |  |  | **3** | -0.003 | 0.006 | 0.617 | -0.017 | 0.011 |
|  |  | **3** | **0** | -.015286435* | 0.006 | 0.041 | -0.030 | -0.001 |
|  |  |  | **1** | -.046691312* | 0.005 | 0.000 | -0.060 | -0.034 |
|  |  |  | **2** | 0.003 | 0.006 | 0.617 | -0.011 | 0.017 |
| ***Granulicella*** | **LSD** | **0** | **1** | -.001969832* | 0.000 | 0.003 | -0.003 | -0.001 |
|  |  |  | **2** | -.014220567* | 0.000 | 0.000 | -0.015 | -0.013 |
|  |  |  | **3** | -.005306853* | 0.000 | 0.000 | -0.006 | -0.004 |
|  |  | **1** | **0** | .001969832* | 0.000 | 0.003 | 0.001 | 0.003 |
|  |  |  | **2** | -.012250735* | 0.000 | 0.000 | -0.013 | -0.011 |
|  |  |  | **3** | -.003337021* | 0.000 | 0.000 | -0.004 | -0.002 |
|  |  | **2** | **0** | .014220567* | 0.000 | 0.000 | 0.013 | 0.015 |
|  |  |  | **1** | .012250735* | 0.000 | 0.000 | 0.011 | 0.013 |
|  |  |  | **3** | .008913714* | 0.000 | 0.000 | 0.008 | 0.010 |
|  |  | **3** | **0** | .005306853* | 0.000 | 0.000 | 0.004 | 0.006 |
|  |  |  | **1** | .003337021* | 0.000 | 0.000 | 0.002 | 0.004 |
|  |  |  | **2** | -.008913714* | 0.000 | 0.000 | -0.010 | -0.008 |
| ***Subgroup13*** | **LSD** | **0** | **1** | 0.000 | 0.000 | 0.590 | -0.001 | 0.001 |
|  |  |  | **2** | -.010820583* | 0.000 | 0.000 | -0.012 | -0.010 |
|  |  |  | **3** | -.003386492* | 0.000 | 0.000 | -0.004 | -0.003 |
|  |  | **1** | **0** | 0.000 | 0.000 | 0.590 | -0.001 | 0.001 |
|  |  |  | **2** | -.011013968* | 0.000 | 0.000 | -0.012 | -0.010 |
|  |  |  | **3** | -.003579877* | 0.000 | 0.000 | -0.004 | -0.003 |
|  |  | **2** | **0** | .010820583* | 0.000 | 0.000 | 0.010 | 0.012 |
|  |  |  | **1** | .011013968* | 0.000 | 0.000 | 0.010 | 0.012 |
|  |  |  | **3** | .007434091* | 0.000 | 0.000 | 0.007 | 0.008 |
|  |  | **3** | **0** | .003386492* | 0.000 | 0.000 | 0.003 | 0.004 |
|  |  |  | **1** | .003579877* | 0.000 | 0.000 | 0.003 | 0.004 |
|  |  |  | **2** | -.007434091* | 0.000 | 0.000 | -0.008 | -0.007 |
| ***Acidothermus*** | **LSD** | **0** | **1** | -.009921116* | 0.002 | 0.002 | -0.015 | -0.005 |
|  |  |  | **2** | 0.000 | 0.002 | 0.843 | -0.004 | 0.005 |
|  |  |  | **3** | 0.000 | 0.002 | 0.896 | -0.005 | 0.005 |
|  |  | **1** | **0** | .009921116* | 0.002 | 0.002 | 0.005 | 0.015 |
|  |  |  | **2** | .010343866* | 0.002 | 0.001 | 0.006 | 0.015 |
|  |  |  | **3** | .010199951* | 0.002 | 0.001 | 0.006 | 0.015 |
|  |  | **2** | **0** | 0.000 | 0.002 | 0.843 | -0.005 | 0.004 |
|  |  |  | **1** | -.010343866* | 0.002 | 0.001 | -0.015 | -0.006 |
|  |  |  | **3** | 0.000 | 0.002 | 0.940 | -0.004 | 0.004 |
|  |  | **3** | **0** | 0.000 | 0.002 | 0.896 | -0.005 | 0.005 |
|  |  |  | **1** | -.010199951* | 0.002 | 0.001 | -0.015 | -0.006 |
|  |  |  | **2** | 0.000 | 0.002 | 0.940 | -0.004 | 0.004 |
| ***Mycobacterium*** | **LSD** | **0** | **1** | .013608930* | 0.002 | 0.001 | 0.008 | 0.019 |
|  |  |  | **2** | 0.000 | 0.003 | 0.956 | -0.006 | 0.007 |
|  |  |  | **3** | .013330095* | 0.002 | 0.001 | 0.008 | 0.019 |
|  |  | **1** | **0** | -.013608930* | 0.002 | 0.001 | -0.019 | -0.008 |
|  |  |  | **2** | -.013460518* | 0.002 | 0.001 | -0.019 | -0.008 |
|  |  |  | **3** | 0.000 | 0.002 | 0.900 | -0.005 | 0.005 |
|  |  | **2** | **0** | 0.000 | 0.003 | 0.956 | -0.007 | 0.006 |
|  |  |  | **1** | .013460518* | 0.002 | 0.001 | 0.008 | 0.019 |
|  |  |  | **3** | .013181683* | 0.002 | 0.001 | 0.007 | 0.019 |
|  |  | **3** | **0** | -.013330095* | 0.002 | 0.001 | -0.019 | -0.008 |
|  |  |  | **1** | 0.000 | 0.002 | 0.900 | -0.005 | 0.005 |
|  |  |  | **2** | -.013181683* | 0.002 | 0.001 | -0.019 | -0.007 |
| ***Streptomyces*** | **LSD** | **0** | **1** | .014247552* | 0.003 | 0.002 | 0.007 | 0.021 |
|  |  |  | **2** | .017773461* | 0.003 | 0.001 | 0.011 | 0.025 |
|  |  |  | **3** | .032290851* | 0.003 | 0.000 | 0.024 | 0.040 |
|  |  | **1** | **0** | -.014247552* | 0.003 | 0.002 | -0.021 | -0.007 |
|  |  |  | **2** | 0.004 | 0.003 | 0.272 | -0.003 | 0.011 |
|  |  |  | **3** | .018043300* | 0.003 | 0.001 | 0.010 | 0.026 |
|  |  | **2** | **0** | -.017773461* | 0.003 | 0.001 | -0.025 | -0.011 |
|  |  |  | **1** | -0.004 | 0.003 | 0.272 | -0.011 | 0.003 |
|  |  |  | **3** | .014517391* | 0.003 | 0.003 | 0.007 | 0.022 |
|  |  | **3** | **0** | -.032290851* | 0.003 | 0.000 | -0.040 | -0.024 |
|  |  |  | **1** | -.018043300* | 0.003 | 0.001 | -0.026 | -0.010 |
|  |  |  | **2** | -.014517391* | 0.003 | 0.003 | -0.022 | -0.007 |
| ***Acetobacteraceae*** | **LSD** | **0** | **1** | -.099957725* | 0.003 | 0.000 | -0.107 | -0.093 |
|  |  |  | **2** | 0.006 | 0.003 | 0.081 | -0.001 | 0.014 |
|  |  |  | **3** | .019167634* | 0.003 | 0.000 | 0.012 | 0.026 |
|  |  | **1** | **0** | .099957725* | 0.003 | 0.000 | 0.093 | 0.107 |
|  |  |  | **2** | .106289970* | 0.003 | 0.000 | 0.099 | 0.114 |
|  |  |  | **3** | .119125359* | 0.003 | 0.000 | 0.112 | 0.126 |
|  |  | **2** | **0** | -0.006 | 0.003 | 0.081 | -0.014 | 0.001 |
|  |  |  | **1** | -.106289970* | 0.003 | 0.000 | -0.114 | -0.099 |
|  |  |  | **3** | .012835389* | 0.003 | 0.004 | 0.006 | 0.020 |
|  |  | **3** | **0** | -.019167634* | 0.003 | 0.000 | -0.026 | -0.012 |
|  |  |  | **1** | -.119125359* | 0.003 | 0.000 | -0.126 | -0.112 |
|  |  |  | **2** | -.012835389* | 0.003 | 0.004 | -0.020 | -0.006 |
| ***Elsterales*** | **LSD** | **0** | **1** | 0.000 | 0.000 | 0.569 | -0.001 | 0.001 |
|  |  |  | **2** | -.002239672* | 0.000 | 0.000 | -0.003 | -0.001 |
|  |  |  | **3** | -.010172967* | 0.000 | 0.000 | -0.011 | -0.009 |
|  |  | **1** | **0** | 0.000 | 0.000 | 0.569 | -0.001 | 0.001 |
|  |  |  | **2** | -.002014805* | 0.000 | 0.001 | -0.003 | -0.001 |
|  |  |  | **3** | -.009948100* | 0.000 | 0.000 | -0.011 | -0.009 |
|  |  | **2** | **0** | .002239672* | 0.000 | 0.000 | 0.001 | 0.003 |
|  |  |  | **1** | .002014805* | 0.000 | 0.001 | 0.001 | 0.003 |
|  |  |  | **3** | -.007933295* | 0.000 | 0.000 | -0.009 | -0.007 |
|  |  | **3** | **0** | .010172967* | 0.000 | 0.000 | 0.009 | 0.011 |
|  |  |  | **1** | .009948100* | 0.000 | 0.000 | 0.009 | 0.011 |
|  |  |  | **2** | .007933295* | 0.000 | 0.000 | 0.007 | 0.009 |
| ***Micropepsaceae*** | **LSD** | **0** | **1** | -0.004 | 0.005 | 0.411 | -0.015 | 0.007 |
|  |  |  | **2** | 0.003 | 0.005 | 0.584 | -0.009 | 0.015 |
|  |  |  | **3** | .064275885* | 0.005 | 0.000 | 0.053 | 0.075 |
|  |  | **1** | **0** | 0.004 | 0.005 | 0.411 | -0.007 | 0.015 |
|  |  |  | **2** | 0.007 | 0.005 | 0.217 | -0.005 | 0.019 |
|  |  |  | **3** | .068305495* | 0.005 | 0.000 | 0.057 | 0.079 |
|  |  | **2** | **0** | -0.003 | 0.005 | 0.584 | -0.015 | 0.009 |
|  |  |  | **1** | -0.007 | 0.005 | 0.217 | -0.019 | 0.005 |
|  |  |  | **3** | .061321137* | 0.005 | 0.000 | 0.049 | 0.073 |
|  |  | **3** | **0** | -.064275885* | 0.005 | 0.000 | -0.075 | -0.053 |
|  |  |  | **1** | -.068305495* | 0.005 | 0.000 | -0.079 | -0.057 |
|  |  |  | **2** | -.061321137* | 0.005 | 0.000 | -0.073 | -0.049 |
| ***Sphingomonas*** | **LSD** | **0** | **1** | .015497810* | 0.002 | 0.000 | 0.011 | 0.020 |
|  |  |  | **2** | -0.004 | 0.002 | 0.138 | -0.009 | 0.002 |
|  |  |  | **3** | .022297777* | 0.002 | 0.000 | 0.017 | 0.027 |
|  |  | **1** | **0** | -.015497810* | 0.002 | 0.000 | -0.020 | -0.011 |
|  |  |  | **2** | -.019356522* | 0.002 | 0.000 | -0.025 | -0.014 |
|  |  |  | **3** | .006799967* | 0.002 | 0.013 | 0.002 | 0.012 |
|  |  | **2** | **0** | 0.004 | 0.002 | 0.138 | -0.002 | 0.009 |
|  |  |  | **1** | .019356522* | 0.002 | 0.000 | 0.014 | 0.025 |
|  |  |  | **3** | .026156489* | 0.002 | 0.000 | 0.021 | 0.032 |
|  |  | **3** | **0** | -.022297777* | 0.002 | 0.000 | -0.027 | -0.017 |
|  |  |  | **1** | -.006799967* | 0.002 | 0.013 | -0.012 | -0.002 |
|  |  |  | **2** | -.026156489* | 0.002 | 0.000 | -0.032 | -0.021 |
| ***Xanthobacteraceae*** | **LSD** | **0** | **1** | -.004029610* | 0.001 | 0.000 | -0.005 | -0.003 |
|  |  |  | **2** | -.007591498* | 0.001 | 0.000 | -0.009 | -0.006 |
|  |  |  | **3** | 0.000 | 0.001 | 0.727 | -0.001 | 0.002 |
|  |  | **1** | **0** | .004029610* | 0.001 | 0.000 | 0.003 | 0.005 |
|  |  |  | **2** | -.003561887* | 0.001 | 0.001 | -0.005 | -0.002 |
|  |  |  | **3** | .004263472* | 0.001 | 0.001 | 0.003 | 0.006 |
|  |  | **2** | **0** | .007591498* | 0.001 | 0.000 | 0.006 | 0.009 |
|  |  |  | **1** | .003561887* | 0.001 | 0.001 | 0.002 | 0.005 |
|  |  |  | **3** | .007825359* | 0.001 | 0.000 | 0.006 | 0.010 |
|  |  | **3** | **0** | 0.000 | 0.001 | 0.727 | -0.002 | 0.001 |
|  |  |  | **1** | -.004263472* | 0.001 | 0.001 | -0.006 | -0.003 |
|  |  |  | **2** | -.007825359* | 0.001 | 0.000 | -0.010 | -0.006 |
| ***Bacillus*** | **LSD** | **0** | **1** | 0.000 | 0.001 | 0.737 | -0.002 | 0.002 |
|  |  |  | **2** | -.002329619* | 0.001 | 0.015 | -0.004 | -0.001 |
|  |  |  | **3** | -.012232746* | 0.001 | 0.000 | -0.014 | -0.011 |
|  |  | **1** | **0** | 0.000 | 0.001 | 0.737 | -0.002 | 0.002 |
|  |  |  | **2** | -.002046287* | 0.001 | 0.039 | -0.004 | 0.000 |
|  |  |  | **3** | -.011949414* | 0.001 | 0.000 | -0.014 | -0.010 |
|  |  | **2** | **0** | .002329619* | 0.001 | 0.015 | 0.001 | 0.004 |
|  |  |  | **1** | .002046287* | 0.001 | 0.039 | 0.000 | 0.004 |
|  |  |  | **3** | -.009903127* | 0.001 | 0.000 | -0.012 | -0.008 |
|  |  | **3** | **0** | .012232746* | 0.001 | 0.000 | 0.011 | 0.014 |
|  |  |  | **1** | .011949414* | 0.001 | 0.000 | 0.010 | 0.014 |
|  |  |  | **2** | .009903127* | 0.001 | 0.000 | 0.008 | 0.012 |
| ***Paenibacillus*** | **LSD** | **0** | **1** | 0.002 | 0.001 | 0.157 | -0.001 | 0.006 |
|  |  |  | **2** | -.013536972* | 0.001 | 0.000 | -0.017 | -0.010 |
|  |  |  | **3** | -0.001 | 0.002 | 0.609 | -0.005 | 0.003 |
|  |  | **1** | **0** | -0.002 | 0.001 | 0.157 | -0.006 | 0.001 |
|  |  |  | **2** | -.015884580* | 0.001 | 0.000 | -0.019 | -0.012 |
|  |  |  | **3** | -0.003 | 0.002 | 0.092 | -0.007 | 0.001 |
|  |  | **2** | **0** | .013536972* | 0.001 | 0.000 | 0.010 | 0.017 |
|  |  |  | **1** | .015884580* | 0.001 | 0.000 | 0.012 | 0.019 |
|  |  |  | **3** | .012650998* | 0.002 | 0.000 | 0.009 | 0.017 |
|  |  | **3** | **0** | 0.001 | 0.002 | 0.609 | -0.003 | 0.005 |
|  |  |  | **1** | 0.003 | 0.002 | 0.092 | -0.001 | 0.007 |
|  |  |  | **2** | -.012650998* | 0.002 | 0.000 | -0.017 | -0.009 |
| ***Sporosarcina*** | **LSD** | **0** | **1** | .038479182* | 0.003 | 0.000 | 0.031 | 0.046 |
|  |  |  | **2** | .027793519* | 0.003 | 0.000 | 0.019 | 0.036 |
|  |  |  | **3** | .031985033* | 0.003 | 0.000 | 0.024 | 0.040 |
|  |  | **1** | **0** | -.038479182* | 0.003 | 0.000 | -0.046 | -0.031 |
|  |  |  | **2** | -.010685663* | 0.003 | 0.014 | -0.018 | -0.003 |
|  |  |  | **3** | -0.006 | 0.003 | 0.058 | -0.013 | 0.000 |
|  |  | **2** | **0** | -.027793519* | 0.003 | 0.000 | -0.036 | -0.019 |
|  |  |  | **1** | .010685663* | 0.003 | 0.014 | 0.003 | 0.018 |
|  |  |  | **3** | 0.004 | 0.003 | 0.225 | -0.003 | 0.012 |
|  |  | **3** | **0** | -.031985033* | 0.003 | 0.000 | -0.040 | -0.024 |
|  |  |  | **1** | 0.006 | 0.003 | 0.058 | 0.000 | 0.013 |
|  |  |  | **2** | -0.004 | 0.003 | 0.225 | -0.012 | 0.003 |
| ***Chitinophagaceae*** | **LSD** | **0** | **1** | .012367666* | 0.001 | 0.000 | 0.010 | 0.015 |
|  |  |  | **2** | .012241740* | 0.001 | 0.000 | 0.010 | 0.015 |
|  |  |  | **3** | .011045450* | 0.001 | 0.000 | 0.009 | 0.013 |
|  |  | **1** | **0** | -.012367666* | 0.001 | 0.000 | -0.015 | -0.010 |
|  |  |  | **2** | 0.000 | 0.001 | 0.903 | -0.002 | 0.002 |
|  |  |  | **3** | -0.001 | 0.001 | 0.225 | -0.004 | 0.001 |
|  |  | **2** | **0** | -.012241740* | 0.001 | 0.000 | -0.015 | -0.010 |
|  |  |  | **1** | 0.000 | 0.001 | 0.903 | -0.002 | 0.002 |
|  |  |  | **3** | -0.001 | 0.001 | 0.268 | -0.004 | 0.001 |
|  |  | **3** | **0** | -.011045450* | 0.001 | 0.000 | -0.013 | -0.009 |
|  |  |  | **1** | 0.001 | 0.001 | 0.225 | -0.001 | 0.004 |
|  |  |  | **2** | 0.001 | 0.001 | 0.268 | -0.001 | 0.004 |
| ***OLB14*** | **LSD** | **0** | **1** | .033909891* | 0.004 | 0.000 | 0.025 | 0.043 |
|  |  |  | **2** | .030680806* | 0.004 | 0.000 | 0.022 | 0.040 |
|  |  |  | **3** | .012169783* | 0.004 | 0.013 | 0.003 | 0.021 |
|  |  | **1** | **0** | -.033909891* | 0.004 | 0.000 | -0.043 | -0.025 |
|  |  |  | **2** | -0.003 | 0.004 | 0.426 | -0.012 | 0.006 |
|  |  |  | **3** | -.021740108* | 0.004 | 0.000 | -0.031 | -0.013 |
|  |  | **2** | **0** | -.030680806* | 0.004 | 0.000 | -0.040 | -0.022 |
|  |  |  | **1** | 0.003 | 0.004 | 0.426 | -0.006 | 0.012 |
|  |  |  | **3** | -.018511023* | 0.004 | 0.001 | -0.027 | -0.010 |
|  |  | **3** | **0** | -.012169783* | 0.004 | 0.013 | -0.021 | -0.003 |
|  |  |  | **1** | .021740108* | 0.004 | 0.000 | 0.013 | 0.031 |
|  |  |  | **2** | .018511023* | 0.004 | 0.001 | 0.010 | 0.027 |
| ***JG30.KF.CM45*** | **LSD** | **0** | **1** | -.012763431* | 0.000 | 0.000 | -0.013 | -0.012 |
|  |  |  | **2** | .004479344* | 0.000 | 0.000 | 0.004 | 0.005 |
|  |  |  | **3** | .002212688* | 0.000 | 0.000 | 0.002 | 0.003 |
|  |  | **1** | **0** | .012763431* | 0.000 | 0.000 | 0.012 | 0.013 |
|  |  |  | **2** | .017242775* | 0.000 | 0.000 | 0.017 | 0.018 |
|  |  |  | **3** | .014976119* | 0.000 | 0.000 | 0.014 | 0.016 |
|  |  | **2** | **0** | -.004479344* | 0.000 | 0.000 | -0.005 | -0.004 |
|  |  |  | **1** | -.017242775* | 0.000 | 0.000 | -0.018 | -0.017 |
|  |  |  | **3** | -.002266656* | 0.000 | 0.000 | -0.003 | -0.002 |
|  |  | **3** | **0** | -.002212688* | 0.000 | 0.000 | -0.003 | -0.002 |
|  |  |  | **1** | -.014976119* | 0.000 | 0.000 | -0.016 | -0.014 |
|  |  |  | **2** | .002266656* | 0.000 | 0.000 | 0.002 | 0.003 |
| ***Nitrolancea*** | **LSD** | **0** | **1** | -.010388840* | 0.001 | 0.000 | -0.012 | -0.009 |
|  |  |  | **2** | .004326434* | 0.001 | 0.001 | 0.003 | 0.006 |
|  |  |  | **3** | .003426967* | 0.001 | 0.002 | 0.002 | 0.005 |
|  |  | **1** | **0** | .010388840* | 0.001 | 0.000 | 0.009 | 0.012 |
|  |  |  | **2** | .014715274* | 0.001 | 0.000 | 0.013 | 0.016 |
|  |  |  | **3** | .013815807* | 0.001 | 0.000 | 0.012 | 0.015 |
|  |  | **2** | **0** | -.004326434* | 0.001 | 0.001 | -0.006 | -0.003 |
|  |  |  | **1** | -.014715274* | 0.001 | 0.000 | -0.016 | -0.013 |
|  |  |  | **3** | -0.001 | 0.001 | 0.213 | -0.002 | 0.001 |
|  |  | **3** | **0** | -.003426967* | 0.001 | 0.002 | -0.005 | -0.002 |
|  |  |  | **1** | -.013815807* | 0.001 | 0.000 | -0.015 | -0.012 |
|  |  |  | **2** | 0.001 | 0.001 | 0.213 | -0.001 | 0.002 |
| ***Acidibacter*** | **LSD** | **0** | **1** | 0.002 | 0.001 | 0.147 | -0.001 | 0.004 |
|  |  |  | **2** | -0.001 | 0.001 | 0.460 | -0.003 | 0.002 |
|  |  |  | **3** | -.024420518* | 0.001 | 0.000 | -0.027 | -0.022 |
|  |  | **1** | **0** | -0.002 | 0.001 | 0.147 | -0.004 | 0.001 |
|  |  |  | **2** | -0.002 | 0.001 | 0.060 | -0.005 | 0.000 |
|  |  |  | **3** | -.026003580* | 0.001 | 0.000 | -0.028 | -0.024 |
|  |  | **2** | **0** | 0.001 | 0.001 | 0.460 | -0.002 | 0.003 |
|  |  |  | **1** | 0.002 | 0.001 | 0.060 | 0.000 | 0.005 |
|  |  |  | **3** | -.023570522* | 0.001 | 0.000 | -0.026 | -0.021 |
|  |  | **3** | **0** | .024420518* | 0.001 | 0.000 | 0.022 | 0.027 |
|  |  |  | **1** | .026003580* | 0.001 | 0.000 | 0.024 | 0.028 |
|  |  |  | **2** | .023570522* | 0.001 | 0.000 | 0.021 | 0.026 |
| ***Chujaibacter*** | **LSD** | **0** | **1** | .013114223* | 0.005 | 0.042 | 0.001 | 0.025 |
|  |  |  | **2** | .024299091* | 0.005 | 0.004 | 0.012 | 0.037 |
|  |  |  | **3** | -0.009 | 0.004 | 0.085 | -0.021 | 0.002 |
|  |  | **1** | **0** | -.013114223* | 0.005 | 0.042 | -0.025 | -0.001 |
|  |  |  | **2** | 0.011 | 0.005 | 0.068 | -0.001 | 0.024 |
|  |  |  | **3** | -.022531639* | 0.004 | 0.004 | -0.034 | -0.011 |
|  |  | **2** | **0** | -.024299091* | 0.005 | 0.004 | -0.037 | -0.012 |
|  |  |  | **1** | -0.011 | 0.005 | 0.068 | -0.024 | 0.001 |
|  |  |  | **3** | -.033716506* | 0.004 | 0.001 | -0.045 | -0.022 |
|  |  | **3** | **0** | 0.009 | 0.004 | 0.085 | -0.002 | 0.021 |
|  |  |  | **1** | .022531639* | 0.004 | 0.004 | 0.011 | 0.034 |
|  |  |  | **2** | .033716507* | 0.004 | 0.001 | 0.022 | 0.045 |
| ***KF.JG30.C25*** | **LSD** | **0** | **1** | .016001511* | 0.002 | 0.000 | 0.012 | 0.020 |
|  |  |  | **2** | .019095676* | 0.002 | 0.000 | 0.015 | 0.023 |
|  |  |  | **3** | .031553289* | 0.002 | 0.000 | 0.028 | 0.035 |
|  |  | **1** | **0** | -.016001511* | 0.002 | 0.000 | -0.020 | -0.012 |
|  |  |  | **2** | 0.003 | 0.002 | 0.086 | -0.001 | 0.007 |
|  |  |  | **3** | .015551778* | 0.002 | 0.000 | 0.012 | 0.019 |
|  |  | **2** | **0** | -.019095676* | 0.002 | 0.000 | -0.023 | -0.015 |
|  |  |  | **1** | -0.003 | 0.002 | 0.086 | -0.007 | 0.001 |
|  |  |  | **3** | .012457613* | 0.002 | 0.000 | 0.009 | 0.016 |
|  |  | **3** | **0** | -.031553289* | 0.002 | 0.000 | -0.035 | -0.028 |
|  |  |  | **1** | -.015551778* | 0.002 | 0.000 | -0.019 | -0.012 |
|  |  |  | **2** | -.012457613* | 0.002 | 0.000 | -0.016 | -0.009 |
| ***Nitrospira*** | **LSD** | **0** | **1** | .024870252* | 0.007 | 0.008 | 0.009 | 0.041 |
|  |  |  | **2** | .042481808* | 0.007 | 0.000 | 0.026 | 0.059 |
|  |  |  | **3** | .088606456* | 0.007 | 0.000 | 0.072 | 0.105 |
|  |  | **1** | **0** | -.024870252* | 0.007 | 0.008 | -0.041 | -0.009 |
|  |  |  | **2** | .017611556* | 0.007 | 0.036 | 0.001 | 0.034 |
|  |  |  | **3** | .063736204* | 0.007 | 0.000 | 0.048 | 0.080 |
|  |  | **2** | **0** | -.042481808* | 0.007 | 0.000 | -0.059 | -0.026 |
|  |  |  | **1** | -.017611556* | 0.007 | 0.036 | -0.034 | -0.001 |
|  |  |  | **3** | .046124648* | 0.007 | 0.000 | 0.030 | 0.062 |
|  |  | **3** | **0** | -.088606456* | 0.007 | 0.000 | -0.105 | -0.072 |
|  |  |  | **1** | -.063736204* | 0.007 | 0.000 | -0.080 | -0.048 |
|  |  |  | **2** | -.046124648* | 0.007 | 0.000 | -0.062 | -0.030 |
| ***Gemmataceae*** | **LSD** | **0** | **1** | -0.015 | 0.008 | 0.094 | -0.033 | 0.003 |
|  |  |  | **2** | -0.007 | 0.008 | 0.400 | -0.025 | 0.011 |
|  |  |  | **3** | .059058978* | 0.008 | 0.000 | 0.041 | 0.077 |
|  |  | **1** | **0** | 0.015 | 0.008 | 0.094 | -0.003 | 0.033 |
|  |  |  | **2** | 0.008 | 0.008 | 0.342 | -0.010 | 0.026 |
|  |  |  | **3** | .074116049* | 0.008 | 0.000 | 0.056 | 0.092 |
|  |  | **2** | **0** | 0.007 | 0.008 | 0.400 | -0.011 | 0.025 |
|  |  |  | **1** | -0.008 | 0.008 | 0.342 | -0.026 | 0.010 |
|  |  |  | **3** | .066110797* | 0.008 | 0.000 | 0.048 | 0.084 |
|  |  | **3** | **0** | -.059058978* | 0.008 | 0.000 | -0.077 | -0.041 |
|  |  |  | **1** | -.074116049* | 0.008 | 0.000 | -0.092 | -0.056 |
|  |  |  | **2** | -.066110797* | 0.008 | 0.000 | -0.084 | -0.048 |
| ***Haliangium*** | **LSD** | **0** | **1** | .012799410* | 0.001 | 0.000 | 0.009 | 0.016 |
|  |  |  | **2** | .012493591* | 0.001 | 0.000 | 0.009 | 0.016 |
|  |  |  | **3** | .011009471* | 0.001 | 0.000 | 0.008 | 0.014 |
|  |  | **1** | **0** | -.012799410* | 0.001 | 0.000 | -0.016 | -0.009 |
|  |  |  | **2** | 0.000 | 0.001 | 0.837 | -0.004 | 0.003 |
|  |  |  | **3** | -0.002 | 0.001 | 0.250 | -0.005 | 0.002 |
|  |  | **2** | **0** | -.012493591* | 0.001 | 0.000 | -0.016 | -0.009 |
|  |  |  | **1** | 0.000 | 0.001 | 0.837 | -0.003 | 0.004 |
|  |  |  | **3** | -0.001 | 0.001 | 0.334 | -0.005 | 0.002 |
|  |  | **3** | **0** | -.011009471* | 0.001 | 0.000 | -0.014 | -0.008 |
|  |  |  | **1** | 0.002 | 0.001 | 0.250 | -0.002 | 0.005 |
|  |  |  | **2** | 0.001 | 0.001 | 0.334 | -0.002 | 0.005 |
| ***Sandaracinaceae*** | **LSD** | **0** | **1** | .018717900* | 0.002 | 0.000 | 0.014 | 0.023 |
|  |  |  | **2** | .033810950* | 0.002 | 0.000 | 0.029 | 0.039 |
|  |  |  | **3** | .033473650* | 0.002 | 0.000 | 0.029 | 0.038 |
|  |  | **1** | **0** | -.018717900* | 0.002 | 0.000 | -0.023 | -0.014 |
|  |  |  | **2** | .015093050* | 0.002 | 0.000 | 0.011 | 0.020 |
|  |  |  | **3** | .014755750* | 0.002 | 0.000 | 0.010 | 0.019 |
|  |  | **2** | **0** | -.033810950* | 0.002 | 0.000 | -0.039 | -0.029 |
|  |  |  | **1** | -.015093050* | 0.002 | 0.000 | -0.020 | -0.011 |
|  |  |  | **3** | 0.000 | 0.002 | 0.865 | -0.005 | 0.004 |
|  |  | **3** | **0** | -.033473650* | 0.002 | 0.000 | -0.038 | -0.029 |
|  |  |  | **1** | -.014755750* | 0.002 | 0.000 | -0.019 | -0.010 |
|  |  |  | **2** | 0.000 | 0.002 | 0.865 | -0.004 | 0.005 |
| ***Saccharimonadales*** | **LSD** | **0** | **1** | -.002630940* | 0.001 | 0.028 | -0.005 | 0.000 |
|  |  |  | **2** | -.008212130* | 0.001 | 0.000 | -0.010 | -0.006 |
|  |  |  | **3** | -.003723792* | 0.001 | 0.004 | -0.006 | -0.002 |
|  |  | **1** | **0** | .002630940* | 0.001 | 0.028 | 0.000 | 0.005 |
|  |  |  | **2** | -.005581190* | 0.001 | 0.001 | -0.008 | -0.004 |
|  |  |  | **3** | -0.001 | 0.001 | 0.237 | -0.003 | 0.001 |
|  |  | **2** | **0** | .008212130* | 0.001 | 0.000 | 0.006 | 0.010 |
|  |  |  | **1** | .005581190* | 0.001 | 0.001 | 0.004 | 0.008 |
|  |  |  | **3** | .004488338* | 0.001 | 0.001 | 0.003 | 0.006 |
|  |  | **3** | **0** | .003723792* | 0.001 | 0.004 | 0.002 | 0.006 |
|  |  |  | **1** | 0.001 | 0.001 | 0.237 | -0.001 | 0.003 |
|  |  |  | **2** | -.004488338* | 0.001 | 0.001 | -0.006 | -0.003 |
| ***Conexibacter*** | **LSD** | **0** | **1** | 0.000 | 0.001 | 0.992 | -0.002 | 0.002 |
|  |  |  | **2** | -0.002 | 0.001 | 0.074 | -0.005 | 0.000 |
|  |  |  | **3** | -.009993074* | 0.001 | 0.000 | -0.012 | -0.008 |
|  |  | **1** | **0** | 0.000 | 0.001 | 0.992 | -0.002 | 0.002 |
|  |  |  | **2** | -0.002 | 0.001 | 0.073 | -0.005 | 0.000 |
|  |  |  | **3** | -.010002069* | 0.001 | 0.000 | -0.012 | -0.008 |
|  |  | **2** | **0** | 0.002 | 0.001 | 0.074 | 0.000 | 0.005 |
|  |  |  | **1** | 0.002 | 0.001 | 0.073 | 0.000 | 0.005 |
|  |  |  | **3** | -.007861338* | 0.001 | 0.000 | -0.010 | -0.005 |
|  |  | **3** | **0** | .009993074* | 0.001 | 0.000 | 0.008 | 0.012 |
|  |  |  | **1** | .010002069* | 0.001 | 0.000 | 0.008 | 0.012 |
|  |  |  | **2** | .007861338* | 0.001 | 0.000 | 0.005 | 0.010 |
| ***Gaiellales*** | **LSD** | **0** | **1** | -0.001 | 0.001 | 0.432 | -0.003 | 0.001 |
|  |  |  | **2** | .002626443* | 0.001 | 0.014 | 0.001 | 0.005 |
|  |  |  | **3** | 0.001 | 0.001 | 0.234 | -0.001 | 0.003 |
|  |  | **1** | **0** | 0.001 | 0.001 | 0.432 | -0.001 | 0.003 |
|  |  |  | **2** | .003350513* | 0.001 | 0.008 | 0.001 | 0.005 |
|  |  |  | **3** | 0.002 | 0.001 | 0.096 | 0.000 | 0.004 |
|  |  | **2** | **0** | -.002626443* | 0.001 | 0.014 | -0.005 | -0.001 |
|  |  |  | **1** | -.003350513* | 0.001 | 0.008 | -0.005 | -0.001 |
|  |  |  | **3** | -0.001 | 0.001 | 0.134 | -0.004 | 0.001 |
|  |  | **3** | **0** | -0.001 | 0.001 | 0.234 | -0.003 | 0.001 |
|  |  |  | **1** | -0.002 | 0.001 | 0.096 | -0.004 | 0.000 |
|  |  |  | **2** | 0.001 | 0.001 | 0.134 | -0.001 | 0.004 |
| ***Solirubrobacteraceae*** | **LSD** | **0** | **1** | 0.001 | 0.002 | 0.720 | -0.003 | 0.004 |
|  |  |  | **2** | .014697285* | 0.002 | 0.000 | 0.011 | 0.018 |
|  |  |  | **3** | .022342751* | 0.002 | 0.000 | 0.019 | 0.026 |
|  |  | **1** | **0** | -0.001 | 0.002 | 0.720 | -0.004 | 0.003 |
|  |  |  | **2** | .014112632* | 0.002 | 0.000 | 0.010 | 0.018 |
|  |  |  | **3** | .021758098* | 0.002 | 0.000 | 0.018 | 0.025 |
|  |  | **2** | **0** | -.014697285* | 0.002 | 0.000 | -0.018 | -0.011 |
|  |  |  | **1** | -.014112632* | 0.002 | 0.000 | -0.018 | -0.010 |
|  |  |  | **3** | .007645466* | 0.002 | 0.001 | 0.004 | 0.011 |
|  |  | **3** | **0** | -.022342751* | 0.002 | 0.000 | -0.026 | -0.019 |
|  |  |  | **1** | -.021758098* | 0.002 | 0.000 | -0.025 | -0.018 |
|  |  |  | **2** | -.007645466* | 0.002 | 0.001 | -0.011 | -0.004 |
| ***Solirubrobacterales*** | **LSD** | **0** | **1** | 0.001 | 0.000 | 0.140 | 0.000 | 0.001 |
|  |  |  | **2** | -.012722955* | 0.000 | 0.000 | -0.014 | -0.012 |
|  |  |  | **3** | -.005486746* | 0.000 | 0.000 | -0.006 | -0.005 |
|  |  | **1** | **0** | -0.001 | 0.000 | 0.140 | -0.001 | 0.000 |
|  |  |  | **2** | -.013235651* | 0.000 | 0.000 | -0.014 | -0.012 |
|  |  |  | **3** | -.005999442* | 0.000 | 0.000 | -0.007 | -0.005 |
|  |  | **2** | **0** | .012722955* | 0.000 | 0.000 | 0.012 | 0.014 |
|  |  |  | **1** | .013235651* | 0.000 | 0.000 | 0.012 | 0.014 |
|  |  |  | **3** | .007236209* | 0.000 | 0.000 | 0.006 | 0.008 |
|  |  | **3** | **0** | .005486746* | 0.000 | 0.000 | 0.005 | 0.006 |
|  |  |  | **1** | .005999442* | 0.000 | 0.000 | 0.005 | 0.007 |
|  |  |  | **2** | -.007236209* | 0.000 | 0.000 | -0.008 | -0.006 |
| ***Vicinamibacterales*** | **LSD** | **0** | **1** | -.015560772* | 0.002 | 0.000 | -0.019 | -0.012 |
|  |  |  | **2** | .005297859* | 0.002 | 0.013 | 0.001 | 0.009 |
|  |  |  | **3** | .005738597* | 0.002 | 0.008 | 0.002 | 0.010 |
|  |  | **1** | **0** | .015560772* | 0.002 | 0.000 | 0.012 | 0.019 |
|  |  |  | **2** | .020858631* | 0.002 | 0.000 | 0.017 | 0.025 |
|  |  |  | **3** | .021299369* | 0.002 | 0.000 | 0.017 | 0.025 |
|  |  | **2** | **0** | -.005297859* | 0.002 | 0.013 | -0.009 | -0.001 |
|  |  |  | **1** | -.020858631* | 0.002 | 0.000 | -0.025 | -0.017 |
|  |  |  | **3** | 0.000 | 0.002 | 0.797 | -0.003 | 0.004 |
|  |  | **3** | **0** | -.005738597* | 0.002 | 0.008 | -0.010 | -0.002 |
|  |  |  | **1** | -.021299369* | 0.002 | 0.000 | -0.025 | -0.017 |
|  |  |  | **2** | 0.000 | 0.002 | 0.797 | -0.004 | 0.003 |
| * The significance level of the difference in the mean value is 0.05. | | | | | | | | |

**Table S5** ANOVA analysis of fungal community on the genus level.

|  | | **Square Sum** | **Variance** | **Mean square** | **F** | **Significance** |
| --- | --- | --- | --- | --- | --- | --- |
| ***Ophiostoma*** | **Within groups** | .202 | 3 | .067 | 153.751 | .000 |
|  | **Between groups** | .004 | 8 | .000 |  |  |
|  | **Total** | .206 | 11 |  |  |  |
| ***Trichoderma*** | **Within groups** | .012 | 3 | .004 | 207.398 | .000 |
|  | **Between groups** | .000 | 6 | .000 |  |  |
|  | **Total** | .012 | 9 |  |  |  |
| ***Acremonium*** | **Within groups** | .011 | 3 | .004 | 429.494 | .000 |
|  | **Between groups** | .000 | 7 | .000 |  |  |
|  | **Total** | .011 | 10 |  |  |  |
| ***Digitaria*** | **Within groups** | .003 | 3 | .001 | 7.607 | .010 |
|  | **Between groups** | .001 | 8 | .000 |  |  |
|  | **Total** | .003 | 11 |  |  |  |
| ***Fusarium*** | **Within groups** | .003 | 3 | .001 | 7.073 | .012 |
|  | **Between groups** | .001 | 8 | .000 |  |  |
|  | **Total** | .005 | 11 |  |  |  |
| ***Colpoda*** | **Within groups** | .011 | 3 | .004 | 125.047 | .000 |
|  | **Between groups** | .000 | 7 | .000 |  |  |
|  | **Total** | .012 | 10 |  |  |  |
| ***Ascotricha*** | **Within groups** | .009 | 3 | .003 | 82.133 | .000 |
|  | **Between groups** | .000 | 6 | .000 |  |  |
|  | **Total** | .010 | 9 |  |  |  |
| ***Stephanonectria*** | **Within groups** | .021 | 3 | .007 | 2164.874 | .000 |
|  | **Between groups** | .000 | 7 | .000 |  |  |
|  | **Total** | .021 | 10 |  |  |  |
| ***Chaetomium*** | **Within groups** | .005 | 3 | .002 | 47.656 | .000 |
|  | **Between groups** | .000 | 6 | .000 |  |  |
|  | **Total** | .005 | 9 |  |  |  |
| ***Nectria*** | **Within groups** | .005 | 3 | .002 | 16.482 | .001 |
|  | **Between groups** | .001 | 8 | .000 |  |  |
|  | **Total** | .005 | 11 |  |  |  |
| ***Entorrhiza*** | **Within groups** | .003 | 3 | .001 | 111.795 | .000 |
|  | **Between groups** | .000 | 7 | .000 |  |  |
|  | **Total** | .003 | 10 |  |  |  |
| ***Thyridariaceae*** | **Within groups** | .002 | 3 | .001 | 36.147 | .000 |
|  | **Between groups** | .000 | 8 | .000 |  |  |
|  | **Total** | .002 | 11 |  |  |  |
| ***Penicillium*** | **Within groups** | .001 | 3 | .000 | 30.521 | .000 |
|  | **Between groups** | .000 | 7 | .000 |  |  |
|  | **Total** | .001 | 10 |  |  |  |
| ***Massarina*** | **Within groups** | .002 | 3 | .001 | 122.041 | .000 |
|  | **Between groups** | .000 | 8 | .000 |  |  |
|  | **Total** | .002 | 11 |  |  |  |

**Table S6** Multiple comparisons analysis of fungal community on the genus level through the LSD method.

|  | | **(I)** | **(J)** | **Mean value (I-J)** | **Standard error** | **Significance** | **95% Confidence interval** | |
| --- | --- | --- | --- | --- | --- | --- | --- | --- |
|  |  | **Group** | **Group** |  |  |  | **Upper limit** | **Lower limit** |
| ***Ophiostoma*** | **LSD** | **0** | **1** | -.309255079* | 0.017 | 0.000 | -0.349 | -0.270 |
|  |  |  | **2** | -0.032 | 0.017 | 0.102 | -0.071 | 0.008 |
|  |  |  | **3** | -0.001 | 0.017 | 0.939 | -0.041 | 0.038 |
|  |  | **1** | **0** | .309255079* | 0.017 | 0.000 | 0.270 | 0.349 |
|  |  |  | **2** | .277652370* | 0.017 | 0.000 | 0.238 | 0.317 |
|  |  |  | **3** | .307909359* | 0.017 | 0.000 | 0.268 | 0.347 |
|  |  | **2** | **0** | 0.032 | 0.017 | 0.102 | -0.008 | 0.071 |
|  |  |  | **1** | -.277652370* | 0.017 | 0.000 | -0.317 | -0.238 |
|  |  |  | **3** | 0.030 | 0.017 | 0.115 | -0.009 | 0.070 |
|  |  | **3** | **0** | 0.001 | 0.017 | 0.939 | -0.038 | 0.041 |
|  |  |  | **1** | -.307909359* | 0.017 | 0.000 | -0.347 | -0.268 |
|  |  |  | **2** | -0.030 | 0.017 | 0.115 | -0.070 | 0.009 |
| ***Trichoderma*** | **LSD** | **0** | **1** | .029880766* | 0.004 | 0.000 | 0.020 | 0.040 |
|  |  |  | **2** | .037962320* | 0.004 | 0.000 | 0.027 | 0.049 |
|  |  |  | **3** | .094547664* | 0.004 | 0.000 | 0.085 | 0.104 |
|  |  | **1** | **0** | -.029880766* | 0.004 | 0.000 | -0.040 | -0.020 |
|  |  |  | **2** | 0.008 | 0.004 | 0.093 | -0.002 | 0.018 |
|  |  |  | **3** | .064666898* | 0.004 | 0.000 | 0.056 | 0.074 |
|  |  | **2** | **0** | -.037962320* | 0.004 | 0.000 | -0.049 | -0.027 |
|  |  |  | **1** | -0.008 | 0.004 | 0.093 | -0.018 | 0.002 |
|  |  |  | **3** | .056585344* | 0.004 | 0.000 | 0.047 | 0.066 |
|  |  | **3** | **0** | -.094547664* | 0.004 | 0.000 | -0.104 | -0.085 |
|  |  |  | **1** | -.064666898* | 0.004 | 0.000 | -0.074 | -0.056 |
|  |  |  | **2** | -.056585344* | 0.004 | 0.000 | -0.066 | -0.047 |
| ***Acremonium*** | **LSD** | **0** | **1** | .087196852* | 0.003 | 0.000 | 0.081 | 0.094 |
|  |  |  | **2** | .061483475* | 0.002 | 0.000 | 0.056 | 0.067 |
|  |  |  | **3** | .063379059* | 0.002 | 0.000 | 0.058 | 0.069 |
|  |  | **1** | **0** | -.087196851* | 0.003 | 0.000 | -0.094 | -0.081 |
|  |  |  | **2** | -.025713376* | 0.003 | 0.000 | -0.032 | -0.019 |
|  |  |  | **3** | -.023817792* | 0.003 | 0.000 | -0.030 | -0.017 |
|  |  | **2** | **0** | -.061483475* | 0.002 | 0.000 | -0.067 | -0.056 |
|  |  |  | **1** | .025713376* | 0.003 | 0.000 | 0.019 | 0.032 |
|  |  |  | **3** | 0.002 | 0.002 | 0.457 | -0.004 | 0.008 |
|  |  | **3** | **0** | -.063379059* | 0.002 | 0.000 | -0.069 | -0.058 |
|  |  |  | **1** | .023817793* | 0.003 | 0.000 | 0.017 | 0.030 |
|  |  |  | **2** | -0.002 | 0.002 | 0.457 | -0.008 | 0.004 |
| ***Digitaria*** | **LSD** | **0** | **1** | 0.011 | 0.009 | 0.241 | -0.009 | 0.031 |
|  |  |  | **2** | .023730971* | 0.009 | 0.024 | 0.004 | 0.044 |
|  |  |  | **3** | .038808821* | 0.009 | 0.002 | 0.019 | 0.059 |
|  |  | **1** | **0** | -0.011 | 0.009 | 0.241 | -0.031 | 0.009 |
|  |  |  | **2** | 0.013 | 0.009 | 0.172 | -0.007 | 0.033 |
|  |  |  | **3** | .027941772* | 0.009 | 0.012 | 0.008 | 0.048 |
|  |  | **2** | **0** | -.023730971* | 0.009 | 0.024 | -0.044 | -0.004 |
|  |  |  | **1** | -0.013 | 0.009 | 0.172 | -0.033 | 0.007 |
|  |  |  | **3** | 0.015 | 0.009 | 0.117 | -0.005 | 0.035 |
|  |  | **3** | **0** | -.038808821* | 0.009 | 0.002 | -0.059 | -0.019 |
|  |  |  | **1** | -.027941772* | 0.009 | 0.012 | -0.048 | -0.008 |
|  |  |  | **2** | -0.015 | 0.009 | 0.117 | -0.035 | 0.005 |
| ***Fusarium*** | **LSD** | **0** | **1** | .029953117* | 0.010 | 0.021 | 0.006 | 0.054 |
|  |  |  | **2** | .030097818* | 0.010 | 0.021 | 0.006 | 0.054 |
|  |  |  | **3** | -0.007 | 0.010 | 0.501 | -0.032 | 0.017 |
|  |  | **1** | **0** | -.029953117* | 0.010 | 0.021 | -0.054 | -0.006 |
|  |  |  | **2** | 0.000 | 0.010 | 0.989 | -0.024 | 0.024 |
|  |  |  | **3** | -.037332870* | 0.010 | 0.007 | -0.061 | -0.013 |
|  |  | **2** | **0** | -.030097818* | 0.010 | 0.021 | -0.054 | -0.006 |
|  |  |  | **1** | 0.000 | 0.010 | 0.989 | -0.024 | 0.024 |
|  |  |  | **3** | -.037477571* | 0.010 | 0.007 | -0.062 | -0.013 |
|  |  | **3** | **0** | 0.007 | 0.010 | 0.501 | -0.017 | 0.032 |
|  |  |  | **1** | .037332870* | 0.010 | 0.007 | 0.013 | 0.061 |
|  |  |  | **2** | .037477571* | 0.010 | 0.007 | 0.013 | 0.062 |
| ***Colpoda*** | **LSD** | **0** | **1** | 0.005 | 0.005 | 0.278 | -0.005 | 0.016 |
|  |  |  | **2** | -.029070441* | 0.005 | 0.001 | -0.041 | -0.017 |
|  |  |  | **3** | -.072625456* | 0.005 | 0.000 | -0.083 | -0.062 |
|  |  | **1** | **0** | -0.005 | 0.005 | 0.278 | -0.016 | 0.005 |
|  |  |  | **2** | -.034380969* | 0.005 | 0.000 | -0.046 | -0.022 |
|  |  |  | **3** | -.077935984* | 0.005 | 0.000 | -0.089 | -0.067 |
|  |  | **2** | **0** | .029070441* | 0.005 | 0.001 | 0.017 | 0.041 |
|  |  |  | **1** | .034380969* | 0.005 | 0.000 | 0.022 | 0.046 |
|  |  |  | **3** | -.043555015* | 0.005 | 0.000 | -0.055 | -0.032 |
|  |  | **3** | **0** | .072625456* | 0.005 | 0.000 | 0.062 | 0.083 |
|  |  |  | **1** | .077935984* | 0.005 | 0.000 | 0.067 | 0.089 |
|  |  |  | **2** | .043555015* | 0.005 | 0.000 | 0.032 | 0.055 |
| ***Ascotricha*** | **LSD** | **0** | **1** | .018123806* | 0.006 | 0.027 | 0.003 | 0.033 |
|  |  |  | **2** | .061910343* | 0.006 | 0.000 | 0.048 | 0.076 |
|  |  |  | **3** | .077292065* | 0.006 | 0.000 | 0.063 | 0.091 |
|  |  | **1** | **0** | -.018123806* | 0.006 | 0.027 | -0.033 | -0.003 |
|  |  |  | **2** | .043786537* | 0.006 | 0.000 | 0.030 | 0.058 |
|  |  |  | **3** | .059168259* | 0.006 | 0.000 | 0.045 | 0.073 |
|  |  | **2** | **0** | -.061910343* | 0.006 | 0.000 | -0.076 | -0.048 |
|  |  |  | **1** | -.043786537* | 0.006 | 0.000 | -0.058 | -0.030 |
|  |  |  | **3** | .015381722* | 0.005 | 0.023 | 0.003 | 0.028 |
|  |  | **3** | **0** | -.077292065* | 0.006 | 0.000 | -0.091 | -0.063 |
|  |  |  | **1** | -.059168259* | 0.006 | 0.000 | -0.073 | -0.045 |
|  |  |  | **2** | -.015381722* | 0.005 | 0.023 | -0.028 | -0.003 |
| ***Stephanonectria*** | **LSD** | **0** | **1** | 0.000 | 0.001 | 0.792 | -0.003 | 0.004 |
|  |  |  | **2** | -0.001 | 0.002 | 0.524 | -0.005 | 0.003 |
|  |  |  | **3** | -.098715055* | 0.001 | 0.000 | -0.102 | -0.095 |
|  |  | **1** | **0** | 0.000 | 0.001 | 0.792 | -0.004 | 0.003 |
|  |  |  | **2** | -0.002 | 0.002 | 0.390 | -0.005 | 0.002 |
|  |  |  | **3** | -.099120218* | 0.001 | 0.000 | -0.103 | -0.096 |
|  |  | **2** | **0** | 0.001 | 0.002 | 0.524 | -0.003 | 0.005 |
|  |  |  | **1** | 0.002 | 0.002 | 0.390 | -0.002 | 0.005 |
|  |  |  | **3** | -.097608092* | 0.002 | 0.000 | -0.102 | -0.094 |
|  |  | **3** | **0** | .098715055* | 0.001 | 0.000 | 0.095 | 0.102 |
|  |  |  | **1** | .099120218* | 0.001 | 0.000 | 0.096 | 0.103 |
|  |  |  | **2** | .097608092* | 0.002 | 0.000 | 0.094 | 0.102 |
| ***Chaetomium*** | **LSD** | **0** | **1** | 0.014 | 0.006 | 0.063 | -0.001 | 0.029 |
|  |  |  | **2** | -.045096082* | 0.006 | 0.000 | -0.059 | -0.032 |
|  |  |  | **3** | -0.002 | 0.006 | 0.736 | -0.015 | 0.012 |
|  |  | **1** | **0** | -0.014 | 0.006 | 0.063 | -0.029 | 0.001 |
|  |  |  | **2** | -.058857151* | 0.006 | 0.000 | -0.072 | -0.045 |
|  |  |  | **3** | -.015707299* | 0.006 | 0.029 | -0.029 | -0.002 |
|  |  | **2** | **0** | .045096082* | 0.006 | 0.000 | 0.032 | 0.059 |
|  |  |  | **1** | .058857151* | 0.006 | 0.000 | 0.045 | 0.072 |
|  |  |  | **3** | .043149853* | 0.005 | 0.000 | 0.031 | 0.055 |
|  |  | **3** | **0** | 0.002 | 0.006 | 0.736 | -0.012 | 0.015 |
|  |  |  | **1** | .015707299* | 0.006 | 0.029 | 0.002 | 0.029 |
|  |  |  | **2** | -.043149853* | 0.005 | 0.000 | -0.055 | -0.031 |
| ***Nectria*** | **LSD** | **0** | **1** | 0.001 | 0.008 | 0.899 | -0.017 | 0.019 |
|  |  |  | **2** | -.046738439* | 0.008 | 0.000 | -0.065 | -0.028 |
|  |  |  | **3** | -.024353187* | 0.008 | 0.015 | -0.043 | -0.006 |
|  |  | **1** | **0** | -0.001 | 0.008 | 0.899 | -0.019 | 0.017 |
|  |  |  | **2** | -.047780286* | 0.008 | 0.000 | -0.066 | -0.030 |
|  |  |  | **3** | -.025395034* | 0.008 | 0.013 | -0.044 | -0.007 |
|  |  | **2** | **0** | .046738439* | 0.008 | 0.000 | 0.028 | 0.065 |
|  |  |  | **1** | .047780286* | 0.008 | 0.000 | 0.030 | 0.066 |
|  |  |  | **3** | .022385252* | 0.008 | 0.022 | 0.004 | 0.041 |
|  |  | **3** | **0** | .024353187* | 0.008 | 0.015 | 0.006 | 0.043 |
|  |  |  | **1** | .025395034* | 0.008 | 0.013 | 0.007 | 0.044 |
|  |  |  | **2** | -.022385252* | 0.008 | 0.022 | -0.041 | -0.004 |
| ***Entorrhiza*** | **LSD** | **0** | **1** | -0.001 | 0.003 | 0.661 | -0.007 | 0.005 |
|  |  |  | **2** | -.030691092* | 0.002 | 0.000 | -0.036 | -0.025 |
|  |  |  | **3** | .007365283* | 0.002 | 0.013 | 0.002 | 0.013 |
|  |  | **1** | **0** | 0.001 | 0.003 | 0.661 | -0.005 | 0.007 |
|  |  |  | **2** | -.029547954* | 0.003 | 0.000 | -0.035 | -0.024 |
|  |  |  | **3** | .008508422* | 0.003 | 0.011 | 0.003 | 0.014 |
|  |  | **2** | **0** | .030691092* | 0.002 | 0.000 | 0.025 | 0.036 |
|  |  |  | **1** | .029547954* | 0.003 | 0.000 | 0.024 | 0.035 |
|  |  |  | **3** | .038056376* | 0.002 | 0.000 | 0.033 | 0.043 |
|  |  | **3** | **0** | -.007365283* | 0.002 | 0.013 | -0.013 | -0.002 |
|  |  |  | **1** | -.008508422* | 0.003 | 0.011 | -0.014 | -0.003 |
|  |  |  | **2** | -.038056376* | 0.002 | 0.000 | -0.043 | -0.033 |
| ***Thyridariaceae*** | **LSD** | **0** | **1** | 0.003 | 0.003 | 0.445 | -0.005 | 0.010 |
|  |  |  | **2** | -0.007 | 0.003 | 0.076 | -0.014 | 0.001 |
|  |  |  | **3** | -.027956242* | 0.003 | 0.000 | -0.035 | -0.020 |
|  |  | **1** | **0** | -0.003 | 0.003 | 0.445 | -0.010 | 0.005 |
|  |  |  | **2** | -.009275337* | 0.003 | 0.022 | -0.017 | -0.002 |
|  |  |  | **3** | -.030575331* | 0.003 | 0.000 | -0.038 | -0.023 |
|  |  | **2** | **0** | 0.007 | 0.003 | 0.076 | -0.001 | 0.014 |
|  |  |  | **1** | .009275337* | 0.003 | 0.022 | 0.002 | 0.017 |
|  |  |  | **3** | -.021299995* | 0.003 | 0.000 | -0.029 | -0.014 |
|  |  | **3** | **0** | .027956242* | 0.003 | 0.000 | 0.020 | 0.035 |
|  |  |  | **1** | .030575331* | 0.003 | 0.000 | 0.023 | 0.038 |
|  |  |  | **2** | .021299995* | 0.003 | 0.000 | 0.014 | 0.029 |
| ***Penicillium*** | **LSD** | **0** | **1** | -.017523297* | 0.003 | 0.000 | -0.024 | -0.011 |
|  |  |  | **2** | -0.003 | 0.003 | 0.275 | -0.010 | 0.003 |
|  |  |  | **3** | -.021690687* | 0.003 | 0.000 | -0.028 | -0.015 |
|  |  | **1** | **0** | .017523297* | 0.003 | 0.000 | 0.011 | 0.024 |
|  |  |  | **2** | .014050471* | 0.003 | 0.002 | 0.007 | 0.021 |
|  |  |  | **3** | -0.004 | 0.003 | 0.156 | -0.010 | 0.002 |
|  |  | **2** | **0** | 0.003 | 0.003 | 0.275 | -0.003 | 0.010 |
|  |  |  | **1** | -.014050471* | 0.003 | 0.002 | -0.021 | -0.007 |
|  |  |  | **3** | -.018217861* | 0.003 | 0.000 | -0.025 | -0.011 |
|  |  | **3** | **0** | .021690687* | 0.003 | 0.000 | 0.015 | 0.028 |
|  |  |  | **1** | 0.004 | 0.003 | 0.156 | -0.002 | 0.010 |
|  |  |  | **2** | .018217862* | 0.003 | 0.000 | 0.011 | 0.025 |
| ***Massarina*** | **LSD** | **0** | **1** | 0.002 | 0.002 | 0.299 | -0.002 | 0.007 |
|  |  |  | **2** | -0.001 | 0.002 | 0.719 | -0.005 | 0.004 |
|  |  |  | **3** | -.030372750* | 0.002 | 0.000 | -0.035 | -0.026 |
|  |  | **1** | **0** | -0.002 | 0.002 | 0.299 | -0.007 | 0.002 |
|  |  |  | **2** | -0.003 | 0.002 | 0.177 | -0.008 | 0.002 |
|  |  |  | **3** | -.032572206* | 0.002 | 0.000 | -0.037 | -0.028 |
|  |  | **2** | **0** | 0.001 | 0.002 | 0.719 | -0.004 | 0.005 |
|  |  |  | **1** | 0.003 | 0.002 | 0.177 | -0.002 | 0.008 |
|  |  |  | **3** | -.029634774* | 0.002 | 0.000 | -0.034 | -0.025 |
|  |  | **3** | **0** | .030372750* | 0.002 | 0.000 | 0.026 | 0.035 |
|  |  |  | **1** | .032572206* | 0.002 | 0.000 | 0.028 | 0.037 |
|  |  |  | **2** | .029634774* | 0.002 | 0.000 | 0.025 | 0.034 |
| * The significance level of the difference in the mean value is 0.05. | | | | | | | | |

**Table S7**correlation analysis between soil properties and representatives of bacterial community.

|  |  | ***Acidimicrobiia*** | ***IMCC26256*** | | ***Acidipila*** | ***Subgroup13*** | ***Acidothermus*** | ***Mycobacterium*** | ***Acetobacteraceae*** | ***OLB14*** | ***JG30.KF.CM45*** | ***Nitrolancea*** | ***Acidibacter*** | ***Chujaibacter*** | ***KF.JG30.C25*** | ***Gemmataceae*** | ***Solirubrobacteraceae*** | ***Vicinamibacterales*** |
| --- | --- | --- | --- | --- | --- | --- | --- | --- | --- | --- | --- | --- | --- | --- | --- | --- | --- | --- |
| **pH** | **Pearson’s coefficient** | -0.628 | -0.742 | | -0.635 | -0.375 | -0.815 | -0.639 | -.988* | -0.607 | .987* | 0.647 | -0.763 | -0.903 | -.978* | -0.575 | -0.653 | 0.936 |
|  | **Significance** | 0.372 | 0.258 | | 0.365 | 0.625 | 0.185 | 0.361 | 0.012 | 0.393 | 0.013 | 0.353 | 0.237 | 0.097 | 0.022 | 0.425 | 0.347 | 0.064 |
| **EC** | **Pearson’s coefficient** | -.961* | -0.447 | | 0.012 | -0.635 | -0.406 | -0.152 | -0.77 | 0.007 | 0.776 | .958* | -0.346 | -0.476 | -0.808 | 0.052 | -0.066 | 0.389 |
|  | **Significance** | 0.039 | 0.553 | | 0.988 | 0.365 | 0.594 | 0.848 | 0.23 | 0.993 | 0.224 | 0.042 | 0.654 | 0.524 | 0.192 | 0.948 | 0.934 | 0.611 |
| **CEC** | **Pearson’s coefficient** | -0.748 | -0.892 | -0.407 | | -0.63 | -0.914 | -0.378 | -.975* | -0.363 | .974* | 0.601 | -0.879 | -.954* | -0.933 | -0.651 | -0.411 | 0.893 |
|  | **Significance** | 0.252 | 0.108 | 0.593 | | 0.37 | 0.086 | 0.622 | 0.025 | 0.637 | 0.026 | 0.399 | 0.121 | 0.046 | 0.067 | 0.349 | 0.589 | 0.107 |
| **AK** | **Pearson’s coefficient** | -0.725 | -0.582 | 0.662 | | -.966* | -0.421 | 0.679 | -0.262 | 0.702 | 0.266 | 0.266 | -0.441 | -0.314 | -0.189 | -0.165 | 0.666 | 0.013 |
|  | **Significance** | 0.275 | 0.418 | 0.338 | | 0.034 | 0.579 | 0.321 | 0.738 | 0.298 | 0.734 | 0.734 | 0.559 | 0.686 | 0.811 | 0.835 | 0.334 | 0.987 |
| **NH4** | **Pearson’s coefficient** | -0.226 | -0.038 | .967* | | -0.596 | 0.146 | .986* | 0.396 | .984* | -0.391 | -0.182 | 0.095 | 0.294 | 0.453 | 0.214 | .982* | -0.574 |
|  | **Significance** | 0.774 | 0.962 | 0.033 | | 0.404 | 0.854 | 0.014 | 0.604 | 0.016 | 0.609 | 0.818 | 0.905 | 0.706 | 0.547 | 0.786 | 0.018 | 0.426 |
| **ROC** | **Pearson’s coefficient** | -0.568 | -0.933 | -0.501 | | -0.543 | -.976* | -0.406 | -0.916 | -0.446 | 0.912 | 0.387 | -.954* | -.999** | -0.85 | -0.819 | -0.479 | .954* |
|  | **Significance** | 0.432 | 0.067 | 0.499 | | 0.457 | 0.024 | 0.594 | 0.084 | 0.554 | 0.088 | 0.613 | 0.046 | 0.001 | 0.15 | 0.181 | 0.521 | 0.046 |
| **Mg** | **Pearson’s coefficient** | 0.447 | .984* | 0.203 | | 0.684 | .972* | 0.025 | 0.671 | 0.128 | -0.667 | -0.061 | .988* | 0.914 | 0.555 | 0.913 | 0.143 | -0.773 |
|  | **Significance** | 0.553 | 0.016 | 0.797 | | 0.316 | 0.028 | 0.975 | 0.329 | 0.872 | 0.333 | 0.939 | 0.012 | 0.086 | 0.445 | 0.087 | 0.857 | 0.227 |
| **Cr** | **Pearson’s coefficient** | -0.162 | 0.693 | 0.475 | | 0.142 | 0.757 | 0.217 | 0.335 | 0.405 | -0.326 | 0.419 | 0.791 | 0.704 | 0.218 | .971* | 0.378 | -0.694 |
|  | **Significance** | 0.838 | 0.307 | 0.525 | | 0.858 | 0.243 | 0.783 | 0.665 | 0.595 | 0.674 | 0.581 | 0.209 | 0.296 | 0.782 | 0.029 | 0.622 | 0.306 |
| **Mn** | **Pearson’s coefficient** | 0.263 | 0.931 | 0.26 | | 0.555 | 0.936 | 0.044 | 0.564 | 0.182 | -0.558 | 0.117 | .962* | 0.87 | 0.439 | .968* | 0.183 | -0.754 |
|  | **Significance** | 0.737 | 0.069 | 0.74 | | 0.445 | 0.064 | 0.956 | 0.436 | 0.818 | 0.442 | 0.883 | 0.038 | 0.13 | 0.561 | 0.032 | 0.817 | 0.246 |
| **Fe** | **Pearson’s coefficient** | 0.003 | 0.537 | 0.925 | | -0.126 | 0.686 | 0.815 | 0.673 | 0.893 | -0.666 | -0.102 | 0.659 | 0.766 | 0.645 | 0.746 | 0.894 | -0.918 |
|  | **Significance** | 0.997 | 0.463 | 0.075 | | 0.874 | 0.314 | 0.185 | 0.327 | 0.107 | 0.334 | 0.898 | 0.341 | 0.234 | 0.355 | 0.254 | 0.106 | 0.082 |
| **Co** | **Pearson’s coefficient** | -0.138 | 0.259 | .997** | | -0.39 | 0.435 | 0.944 | 0.548 | .989* | -0.542 | -0.126 | 0.396 | 0.553 | 0.561 | 0.513 | .986* | -0.781 |
|  | **Significance** | 0.862 | 0.741 | 0.003 | | 0.61 | 0.565 | 0.056 | 0.452 | 0.011 | 0.458 | 0.874 | 0.604 | 0.447 | 0.439 | 0.487 | 0.014 | 0.219 |
| **Ni** | **Pearson’s coefficient** | -0.743 | 0.079 | 0.203 | | -0.319 | 0.129 | -0.057 | -0.378 | 0.159 | 0.386 | 0.913 | 0.194 | 0.04 | -0.474 | 0.562 | 0.093 | -0.075 |
|  | **Significance** | 0.257 | 0.921 | 0.797 | | 0.681 | 0.871 | 0.943 | 0.622 | 0.841 | 0.614 | 0.087 | 0.806 | 0.96 | 0.526 | 0.438 | 0.907 | 0.925 |
| **Tl** | **Pearson’s coefficient** | 0.601 | .992** | 0.3 | | 0.695 | .994** | 0.178 | 0.836 | 0.235 | -0.833 | -0.3 | .988* | .976* | 0.747 | 0.842 | 0.265 | -0.862 |
|  | **Significance** | 0.399 | 0.008 | 0.7 | | 0.305 | 0.006 | 0.822 | 0.164 | 0.765 | 0.167 | 0.7 | 0.012 | 0.024 | 0.253 | 0.158 | 0.735 | 0.138 |
| **SDH** | **Pearson’s coefficient** | -0.718 | -0.924 | -0.395 | | -0.645 | -0.944 | -0.344 | -.956* | -0.346 | .954* | 0.537 | -0.914 | -.972* | -0.902 | -0.705 | -0.39 | 0.9 |
|  | **Significance** | 0.282 | 0.076 | 0.605 | | 0.355 | 0.056 | 0.656 | 0.044 | 0.654 | 0.046 | 0.463 | 0.086 | 0.028 | 0.098 | 0.295 | 0.61 | 0.1 |
| **SC** | **Pearson’s coefficient** | -0.727 | 0.039 | 0.008 | | -0.24 | 0.055 | -0.248 | -0.489 | -0.035 | 0.497 | .958* | 0.129 | -0.06 | -0.592 | 0.481 | -0.102 | 0.072 |
|  | **Significance** | 0.273 | 0.961 | 0.992 | | 0.76 | 0.945 | 0.752 | 0.511 | 0.965 | 0.503 | 0.042 | 0.871 | 0.94 | 0.408 | 0.519 | 0.898 | 0.928 |
| **UR** | **Pearson’s coefficient** | -0.77 | -0.785 | -0.466 | | -0.54 | -0.823 | -0.488 | -.999** | -0.437 | .999** | 0.724 | -0.772 | -0.894 | -.985* | -0.52 | -0.491 | 0.872 |
|  | **Significance** | 0.23 | 0.215 | 0.534 | | 0.46 | 0.177 | 0.512 | 0.001 | 0.563 | 0.001 | 0.276 | 0.228 | 0.106 | 0.015 | 0.48 | 0.509 | 0.128 |
| * At the 0.05 level (two tailed), the correlation is significant. | | | | | | | | | | | | | | | | | | |
| ** At the 0.01 level (two tailed), the correlation is significant. | | | | | | | | | | | | | | | | | | |

**Table S8** Correlation analysis between soil properties and representatives of fungal community.

|  |  | ***Colpoda*** | ***Massarina*** | ***Thyridariaceae*** | ***Entorrhiza*** | ***Digitaria*** | ***Ophiostoma*** | ***Stephanonectria*** | ***Trichoderma*** |
| --- | --- | --- | --- | --- | --- | --- | --- | --- | --- |
| **pH** | **Pearson’s coefficient** | .973* | .986* | .997** | .983* | -0.864 | -0.548 | .973* | -0.902 |
|  | **Significance** | 0.027 | 0.014 | 0.003 | 0.017 | 0.136 | 0.452 | 0.027 | 0.098 |
| **EC** | **Pearson’s coefficient** | 0.497 | 0.78 | 0.627 | 0.576 | -0.34 | -0.148 | 0.804 | -0.554 |
|  | **Significance** | 0.503 | 0.22 | 0.373 | 0.424 | 0.66 | 0.852 | 0.196 | 0.446 |
| **CEC** | **Pearson’s coefficient** | 0.924 | .973* | .957* | .964* | -0.9 | -0.268 | .980* | -.974* |
|  | **Significance** | 0.076 | 0.027 | 0.043 | 0.036 | 0.1 | 0.732 | 0.02 | 0.026 |
| **AK** | **Pearson’s coefficient** | 0.041 | 0.265 | 0.124 | 0.158 | -0.214 | 0.733 | 0.331 | -0.407 |
|  | **Significance** | 0.959 | 0.735 | 0.876 | 0.842 | 0.786 | 0.267 | 0.669 | 0.593 |
| **NH4** | **Pearson’s coefficient** | -0.574 | -0.392 | -0.518 | -0.48 | 0.356 | .977* | -0.327 | 0.214 |
|  | **Significance** | 0.426 | 0.608 | 0.482 | 0.52 | 0.644 | 0.023 | 0.673 | 0.786 |
| **ROC** | **Pearson’s coefficient** | .952* | 0.909 | 0.945 | .973* | -.982* | -0.272 | 0.91 | -.995** |
|  | **Significance** | 0.048 | 0.091 | 0.055 | 0.027 | 0.018 | 0.728 | 0.09 | 0.005 |
| **Mg** | **Pearson’s coefficient** | -0.734 | -0.661 | -0.705 | -0.771 | 0.914 | -0.131 | -0.681 | 0.916 |
|  | **Significance** | 0.266 | 0.339 | 0.295 | 0.229 | 0.086 | 0.869 | 0.319 | 0.084 |
| **Cr** | **Pearson’s coefficient** | -0.597 | -0.318 | -0.481 | -0.563 | 0.796 | 0.067 | -0.307 | 0.64 |
|  | **Significance** | 0.403 | 0.682 | 0.519 | 0.437 | 0.204 | 0.933 | 0.693 | 0.36 |
| **Mn** | **Pearson’s coefficient** | -0.692 | -0.551 | -0.633 | -0.71 | 0.899 | -0.118 | -0.564 | 0.852 |
|  | **Significance** | 0.308 | 0.449 | 0.367 | 0.29 | 0.101 | 0.882 | 0.436 | 0.148 |
| **Fe** | **Pearson’s coefficient** | -0.884 | -0.662 | -0.808 | -0.822 | 0.829 | 0.712 | -0.619 | 0.694 |
|  | **Significance** | 0.116 | 0.338 | 0.192 | 0.178 | 0.171 | 0.288 | 0.381 | 0.306 |
| **Co** | **Pearson’s coefficient** | -0.76 | -0.54 | -0.687 | -0.676 | 0.622 | 0.884 | -0.483 | 0.471 |
|  | **Significance** | 0.24 | 0.46 | 0.313 | 0.324 | 0.378 | 0.116 | 0.517 | 0.529 |
| **Ni** | **Pearson’s coefficient** | 0.054 | 0.394 | 0.206 | 0.122 | 0.181 | -0.137 | 0.412 | -0.044 |
|  | **Significance** | 0.946 | 0.606 | 0.794 | 0.878 | 0.819 | 0.863 | 0.588 | 0.956 |
| **Tl** | **Pearson’s coefficient** | -0.852 | -0.829 | -0.848 | -0.892 | .952* | 0.035 | -0.845 | .986* |
|  | **Significance** | 0.148 | 0.171 | 0.152 | 0.108 | 0.048 | 0.965 | 0.155 | 0.014 |
| **SDH** | **Pearson’s coefficient** | 0.922 | .952* | 0.946 | .961* | -0.925 | -0.225 | .961* | -.989* |
|  | **Significance** | 0.078 | 0.048 | 0.054 | 0.039 | 0.075 | 0.775 | 0.039 | 0.011 |
| **SC** | **Pearson’s coefficient** | 0.201 | 0.505 | 0.342 | 0.252 | 0.07 | -0.32 | 0.511 | -0.13 |
|  | **Significance** | 0.799 | 0.495 | 0.658 | 0.748 | 0.93 | 0.68 | 0.489 | 0.87 |
| **UR** | **Pearson’s coefficient** | 0.922 | .999** | .970* | .956* | -0.83 | -0.401 | 1.000** | -0.916 |
|  | **Significance** | 0.078 | 0.001 | 0.03 | 0.044 | 0.17 | 0.599 | 0 | 0.084 |
| * At the 0.05 level (two tailed), the correlation is significant; ** At the 0.01 level (two tailed), the correlation is significant. | | | | | | | | | |

**Table S9** Correlation analysis between soil properties and diversity of bacterial and fungal communities.

|  | **Soil property** | **Correlation coefficient** | **P-value** |
| --- | --- | --- | --- |
| **Bacteria** | **pH** | 0.539385752 | 0.001 |
|  | **EC** | 0.645720129 | 0.001 |
|  | **AK** | 0.480152385 | 0.002 |
|  | **CEC** | 0.34821157 | 0.003 |
|  | **NH4** | 0.355271611 | 0.005 |
|  | **ROC** | 0.53884956 | 0.001 |
|  | **SDH** | 0.375300378 | 0.001 |
|  | **SC** | 0.481359391 | 0.001 |
|  | **UR** | 0.480017893 | 0.001 |
|  | **Mg** | 0.076604197 | 0.143 |
|  | **Cr** | 0.454956078 | 0.001 |
|  | **Mn** | 0.346108884 | 0.007 |
|  | **Fe** | 0.588697662 | 0.001 |
|  | **Co** | 0.387751629 | 0.003 |
|  | **Ni** | 0.425532316 | 0.002 |
|  | **Tl** | 0.248846668 | 0.001 |
| **Fungi** | **pH** | 0.593860183 | 0.001 |
|  | **EC** | 0.484012687 | 0.002 |
|  | **AK** | 0.46918229 | 0.004 |
|  | **CEC** | 0.357006543 | 0.007 |
|  | **NH4** | 0.446008133 | 0.004 |
|  | **ROC** | 0.610671477 | 0.001 |
|  | **SDH** | 0.388251265 | 0.001 |
|  | **SC** | 0.335279815 | 0.009 |
|  | **UR** | 0.511816441 | 0.001 |
|  | **Mg** | 0.164252931 | 0.016 |
|  | **Cr** | 0.454967545 | 0.003 |
|  | **Mn** | 0.445801828 | 0.001 |
|  | **Fe** | 0.684057013 | 0.001 |
|  | **Co** | 0.424564492 | 0.001 |
|  | **Ni** | 0.231468916 | 0.037 |
|  | **Tl** | 0.288978952 | 0.001 |

**Table S10** Correlation analysis of soil properties

|  | **pH** | **EC** | **AK** | **CEC** | **NH4** | **ROC** | **SDH** | **SC** | **UR** | **Mg** | **Cr** | **Mn** | **Fe** | **Co** | **Ni** | **Tl** |
| --- | --- | --- | --- | --- | --- | --- | --- | --- | --- | --- | --- | --- | --- | --- | --- | --- |
| **pH** | 1.00 |  |  |  |  |  |  |  |  |  |  |  |  |  |  |  |
| **EC** | 0.67  * | 1.00 |  |  |  |  |  |  |  |  |  |  |  |  |  |  |
| **AK** | 0.12 | 0.51 | 1.00 |  |  |  |  |  |  |  |  |  |  |  |  |  |
| **CEC** | 0.80  ** | 0.58  * | 0.36 | 1.00 |  |  |  |  |  |  |  |  |  |  |  |  |
| **NH4** | -0.51 | 0.02 | 0.76  ** | -0.22 | 1.00 |  |  |  |  |  |  |  |  |  |  |  |
| **ROC** | 0.90  *** | 0.50 | 0.29 | 0.82  ** | -0.28 | 1.00 |  |  |  |  |  |  |  |  |  |  |
| **SDH** | 0.84  *** | 0.61  * | 0.35 | 0.62  * | -0.19 | 0.83  *** | 1.00 |  |  |  |  |  |  |  |  |  |
| **SC** | 0.42 | 0.83  *** | 0.16 | 0.24 | -0.10 | 0.13 | 0.23 | 1.00 |  |  |  |  |  |  |  |  |
| **UR** | 0.97  *** | 0.79  ** | 0.31 | 0.80  ** | -0.35 | 0.87  *** | 0.88  *** | 0.47 | 1.00 |  |  |  |  |  |  |  |
| **Mg** | -0.42 | -0.13 | -0.40 | -0.34 | -0.11 | -0.61  * | -0.49 | 0.24 | -0.40 | 1.00 |  |  |  |  |  |  |
| **Cr** | -0.42 | 0.25 | 0.01 | -0.34 | 0.22 | -0.65  * | -0.51 | 0.53 | -0.34 | 0.53 | 1.00 |  |  |  |  |  |
| **Mn** | -0.56 | -0.10 | -0.37 | -0.51 | -0.01 | -0.80  ** | -0.66  * | 0.32 | -0.54 | 0.90  *** | 0.81  ** | 1.00 |  |  |  |  |
| **Fe** | -0.77  ** | -0.08 | 0.36 | -0.49 | 0.79  ** | -0.72  ** | -0.62  * | 0.10 | -0.64  * | 0.40 | 0.70  * | 0.58 | 1.00 |  |  |  |
| **Co** | -0.61  * | -0.01 | 0.55 | -0.32 | 0.79  ** | -0.54 | -0.41 | -0.02 | -0.42 | 0.11 | 0.50 | 0.28  * | 0.83  *** | 1.00 |  |  |
| **Ni** | 0.27 | 0.82  *** | 0.31 | 0.19 | 0.06 | -0.03 | 0.13 | 0.93  *** | 0.38 | 0.25 | 0.70  * | 0.40 | 0.28 | 0.23 | 1.00 | \|  \| \| --- \| |
| **Tl** | -0.70  * | -0.39 | -0.37 | -0.61  * | 0.06 | -0.83  *** | -0.72  ** | -0.10 | -0.65  * | 0.59  * | 0.59  * | 0.71  ** | 0.53 | 0.54 | 0.05 | 1.00 |

*P < 0.05, ** P < 0.01 and ***P < 0.001

**Table S11** Correlation analysis of soil bacteria.

|  |  | IMCC26256 | Acidipila | Bryobacter | Subgroup13 | Acidothermus | Mycobacterium | Acetobacteraceae | Elsterales | OLB14 | JG30.KF.CM45 | Acidibacter | Chujaibacter | KF.JG30.C25 | Conexibacter | Gaiellales | Solirubrobacteraceae | Vicinamibacterales |
| --- | --- | --- | --- | --- | --- | --- | --- | --- | --- | --- | --- | --- | --- | --- | --- | --- | --- | --- |
| Acidimicrobiia | Pearson's analysis | 0.594 | -0.171 | 0.55 | 0.823 | 0.517 | -0.058 | 0.741 | 0.941 | -0.184 | -0.746 | 0.475 | 0.539 | 0.742 | 0.274 | 0.485 | -0.115 | -0.377 |
|  | Significance | 0.406 | 0.829 | 0.45 | 0.177 | 0.483 | 0.942 | 0.259 | 0.059 | 0.816 | 0.254 | 0.525 | 0.461 | 0.258 | 0.726 | 0.515 | 0.885 | 0.623 |
| IMCC26256 | Pearson's analysis | 1 | 0.192 | -0.311 | 0.752 | .982* | 0.056 | 0.77 | 0.762 | 0.123 | -0.768 | .985* | 0.942 | 0.67 | 0.781 | -0.315 | 0.15 | -0.795 |
|  | Significance |  | 0.808 | 0.689 | 0.248 | 0.018 | 0.944 | 0.23 | 0.238 | 0.877 | 0.232 | 0.015 | 0.058 | 0.33 | 0.219 | 0.685 | 0.85 | 0.205 |
| Acidipila | Pearson's analysis | 0.192 | 1 | -0.623 | -0.446 | 0.372 | .959* | 0.51 | -0.276 | .997** | -0.504 | 0.33 | 0.496 | 0.532 | 0.763 | -0.768 | .993** | -0.739 |
|  | Significance | 0.808 |  | 0.377 | 0.554 | 0.628 | 0.041 | 0.49 | 0.724 | 0.003 | 0.496 | 0.67 | 0.504 | 0.468 | 0.237 | 0.232 | 0.007 | 0.261 |
| Acidobacteriaceae | Pearson's analysis | 0.285 | 0.844 | -0.155 | -0.129 | 0.421 | 0.924 | 0.788 | 0.124 | 0.854 | -0.787 | 0.349 | 0.578 | 0.844 | 0.719 | -0.347 | 0.889 | -0.761 |
|  | Significance | 0.715 | 0.156 | 0.845 | 0.871 | 0.579 | 0.076 | 0.212 | 0.876 | 0.146 | 0.213 | 0.651 | 0.422 | 0.156 | 0.281 | 0.653 | 0.111 | 0.239 |
| Bryobacter | Pearson's analysis | -0.311 | -0.623 | 1 | 0.315 | -0.426 | -0.382 | -0.058 | 0.376 | -0.576 | 0.048 | -0.458 | -0.404 | 0.024 | -0.611 | .979* | -0.529 | 0.512 |
|  | Significance | 0.689 | 0.377 |  | 0.685 | 0.574 | 0.618 | 0.942 | 0.624 | 0.424 | 0.952 | 0.542 | 0.596 | 0.976 | 0.389 | 0.021 | 0.471 | 0.488 |
| Subgroup13 | Pearson's analysis | 0.752 | -0.446 | 0.315 | 1 | 0.622 | -0.471 | 0.497 | .962* | -0.493 | -0.5 | 0.63 | 0.541 | 0.422 | 0.209 | 0.362 | -0.449 | -0.267 |
|  | Significance | 0.248 | 0.554 | 0.685 |  | 0.378 | 0.529 | 0.503 | 0.038 | 0.507 | 0.5 | 0.37 | 0.459 | 0.578 | 0.791 | 0.638 | 0.551 | 0.733 |
| Acidothermus | Pearson's analysis | .982* | 0.372 | -0.426 | 0.622 | 1 | 0.231 | 0.817 | 0.66 | 0.305 | -0.813 | .996** | .984* | 0.724 | 0.884 | -0.455 | 0.329 | -0.891 |
|  | Significance | 0.018 | 0.628 | 0.574 | 0.378 |  | 0.769 | 0.183 | 0.34 | 0.695 | 0.187 | 0.004 | 0.016 | 0.276 | 0.116 | 0.545 | 0.671 | 0.109 |
| Mycobacterium | Pearson's analysis | 0.056 | .959* | -0.382 | -0.471 | 0.231 | 1 | 0.53 | -0.251 | .975* | -0.527 | 0.172 | 0.388 | 0.591 | 0.644 | -0.559 | .985* | -0.648 |
|  | Significance | 0.944 | 0.041 | 0.618 | 0.529 | 0.769 |  | 0.47 | 0.749 | 0.025 | 0.473 | 0.828 | 0.612 | 0.409 | 0.356 | 0.441 | 0.015 | 0.352 |
| Acetobacteraceae | Pearson's analysis | 0.77 | 0.51 | -0.058 | 0.497 | 0.817 | 0.53 | 1 | 0.68 | 0.482 | -1.000** | 0.765 | 0.895 | .989* | 0.826 | -0.188 | 0.535 | -0.887 |
|  | Significance | 0.23 | 0.49 | 0.942 | 0.503 | 0.183 | 0.47 |  | 0.32 | 0.518 | 0 | 0.235 | 0.105 | 0.011 | 0.174 | 0.812 | 0.465 | 0.113 |
| Elsterales | Pearson's analysis | 0.762 | -0.276 | 0.376 | .962* | 0.66 | -0.251 | 0.68 | 1 | -0.313 | -0.684 | 0.645 | 0.626 | 0.632 | 0.32 | 0.369 | -0.256 | -0.399 |
|  | Significance | 0.238 | 0.724 | 0.624 | 0.038 | 0.34 | 0.749 | 0.32 |  | 0.687 | 0.316 | 0.355 | 0.374 | 0.368 | 0.68 | 0.631 | 0.744 | 0.601 |
| Xanthobacteraceae | Pearson's analysis | -0.007 | -0.673 | 0.947 | 0.603 | -0.146 | -0.479 | 0.121 | 0.643 | -0.65 | -0.131 | -0.171 | -0.155 | 0.165 | -0.441 | 0.945 | -0.595 | 0.338 |
|  | Significance | 0.993 | 0.327 | 0.053 | 0.397 | 0.854 | 0.521 | 0.879 | 0.357 | 0.35 | 0.869 | 0.829 | 0.845 | 0.835 | 0.559 | 0.055 | 0.405 | 0.662 |
| Bacillus | Pearson's analysis | 0.815 | -0.229 | -0.352 | 0.734 | 0.734 | -0.441 | 0.261 | 0.583 | -0.306 | -0.257 | 0.785 | 0.6 | 0.12 | 0.396 | -0.238 | -0.309 | -0.37 |
|  | Significance | 0.185 | 0.771 | 0.648 | 0.266 | 0.266 | 0.559 | 0.739 | 0.417 | 0.694 | 0.743 | 0.215 | 0.4 | 0.88 | 0.604 | 0.762 | 0.691 | 0.63 |
| Sporosarcina | Pearson's analysis | 0.363 | -0.33 | -0.514 | 0.292 | 0.295 | -0.583 | -0.306 | 0.046 | -0.393 | 0.312 | 0.375 | 0.13 | -0.443 | 0.039 | -0.339 | -0.436 | 0.041 |
|  | Significance | 0.637 | 0.67 | 0.486 | 0.708 | 0.705 | 0.417 | 0.694 | 0.954 | 0.607 | 0.688 | 0.625 | 0.87 | 0.557 | 0.961 | 0.661 | 0.564 | 0.959 |
| OLB14 | Pearson's analysis | 0.123 | .997** | -0.576 | -0.493 | 0.305 | .975* | 0.482 | -0.313 | 1 | -0.477 | 0.26 | 0.438 | 0.516 | 0.715 | -0.728 | .997** | -0.694 |
|  | Significance | 0.877 | 0.003 | 0.424 | 0.507 | 0.695 | 0.025 | 0.518 | 0.687 |  | 0.523 | 0.74 | 0.562 | 0.484 | 0.285 | 0.272 | 0.003 | 0.306 |
| JG30.KF.CM45 | Pearson's analysis | -0.768 | -0.504 | 0.048 | -0.5 | -0.813 | -0.527 | -1.000** | -0.684 | -0.477 | 1 | -0.761 | -0.891 | -.990* | -0.82 | 0.178 | -0.53 | 0.883 |
|  | Significance | 0.232 | 0.496 | 0.952 | 0.5 | 0.187 | 0.473 | 0 | 0.316 | 0.523 |  | 0.239 | 0.109 | 0.01 | 0.18 | 0.822 | 0.47 | 0.117 |
| Nitrolancea | Pearson's analysis | -0.236 | -0.126 | -0.613 | -0.388 | -0.23 | -0.336 | -0.715 | -0.613 | -0.15 | 0.722 | -0.156 | -0.342 | -0.79 | -0.224 | -0.464 | -0.221 | 0.337 |
|  | Significance | 0.764 | 0.874 | 0.387 | 0.612 | 0.77 | 0.664 | 0.285 | 0.387 | 0.85 | 0.278 | 0.844 | 0.658 | 0.21 | 0.776 | 0.536 | 0.779 | 0.663 |
| Acidibacter | Pearson's analysis | .985* | 0.33 | -0.458 | 0.63 | .996** | 0.172 | 0.765 | 0.645 | 0.26 | -0.761 | 1 | .966* | 0.663 | 0.861 | -0.471 | 0.28 | -0.86 |
|  | Significance | 0.015 | 0.67 | 0.542 | 0.37 | 0.004 | 0.828 | 0.235 | 0.355 | 0.74 | 0.239 |  | 0.034 | 0.337 | 0.139 | 0.529 | 0.72 | 0.14 |
| Chujaibacter | Pearson's analysis | 0.942 | 0.496 | -0.404 | 0.541 | .984* | 0.388 | 0.895 | 0.626 | 0.438 | -0.891 | .966* | 1 | 0.823 | 0.935 | -0.469 | 0.468 | -.951* |
|  | Significance | 0.058 | 0.504 | 0.596 | 0.459 | 0.016 | 0.612 | 0.105 | 0.374 | 0.562 | 0.109 | 0.034 |  | 0.177 | 0.065 | 0.531 | 0.532 | 0.049 |
| KF.JG30.C25 | Pearson's analysis | 0.67 | 0.532 | 0.024 | 0.422 | 0.724 | 0.591 | .989* | 0.632 | 0.516 | -.990* | 0.663 | 0.823 | 1 | 0.773 | -0.126 | 0.572 | -0.842 |
|  | Significance | 0.33 | 0.468 | 0.976 | 0.578 | 0.276 | 0.409 | 0.011 | 0.368 | 0.484 | 0.01 | 0.337 | 0.177 |  | 0.227 | 0.874 | 0.428 | 0.158 |
| Gemmataceae | Pearson's analysis | 0.842 | 0.455 | -0.774 | 0.333 | 0.891 | 0.223 | 0.526 | 0.296 | 0.381 | -0.518 | 0.914 | 0.846 | 0.409 | 0.85 | -0.758 | 0.371 | -0.802 |
|  | Significance | 0.158 | 0.545 | 0.226 | 0.667 | 0.109 | 0.777 | 0.474 | 0.704 | 0.619 | 0.482 | 0.086 | 0.154 | 0.591 | 0.15 | 0.242 | 0.629 | 0.198 |
| Conexibacter | Pearson's analysis | 0.781 | 0.763 | -0.611 | 0.209 | 0.884 | 0.644 | 0.826 | 0.32 | 0.715 | -0.82 | 0.861 | 0.935 | 0.773 | 1 | -0.704 | 0.73 | -.993** |
|  | Significance | 0.219 | 0.237 | 0.389 | 0.791 | 0.116 | 0.356 | 0.174 | 0.68 | 0.285 | 0.18 | 0.139 | 0.065 | 0.227 |  | 0.296 | 0.27 | 0.007 |
| Gaiellales | Pearson's analysis | -0.315 | -0.768 | .979* | 0.362 | -0.455 | -0.559 | -0.188 | 0.369 | -0.728 | 0.178 | -0.471 | -0.469 | -0.126 | -0.704 | 1 | -0.689 | 0.617 |
|  | Significance | 0.685 | 0.232 | 0.021 | 0.638 | 0.545 | 0.441 | 0.812 | 0.631 | 0.272 | 0.822 | 0.529 | 0.531 | 0.874 | 0.296 |  | 0.311 | 0.383 |
| Solirubrobacteraceae | Pearson's analysis | 0.15 | .993** | -0.529 | -0.449 | 0.329 | .985* | 0.535 | -0.256 | .997** | -0.53 | 0.28 | 0.468 | 0.572 | 0.73 | -0.689 | 1 | -0.717 |
|  | Significance | 0.85 | 0.007 | 0.471 | 0.551 | 0.671 | 0.015 | 0.465 | 0.744 | 0.003 | 0.47 | 0.72 | 0.532 | 0.428 | 0.27 | 0.311 |  | 0.283 |
| Vicinamibacterales | Pearson's analysis | -0.795 | -0.739 | 0.512 | -0.267 | -0.891 | -0.648 | -0.887 | -0.399 | -0.694 | 0.883 | -0.86 | -.951* | -0.842 | -.993** | 0.617 | -0.717 | 1 |
|  | Significance | 0.205 | 0.261 | 0.488 | 0.733 | 0.109 | 0.352 | 0.113 | 0.601 | 0.306 | 0.117 | 0.14 | 0.049 | 0.158 | 0.007 | 0.383 | 0.283 |  |
| *(P<0.05) |  |  |  |  |  |  |  |  |  |  |  |  |  |  |  |  |  |  |
| **(P<0.01) |  |  |  |  |  |  |  |  |  |  |  |  |  |  |  |  |  |  |

**Table S12** Correlation analysis of soil fungi.

|  |  | Colpoda | Massarina | Thyridariaceae | Entorrhiza | Digitaria | Ascotricha | Stephanonectria | Trichoderma |
| --- | --- | --- | --- | --- | --- | --- | --- | --- | --- |
| Colpoda | Pearson's analysis | 1 | 0.93 | .987* | .992** | -0.938 | -0.907 | 0.911 | -0.921 |
|  | Significance |  | 0.07 | 0.013 | 0.008 | 0.062 | 0.093 | 0.089 | 0.079 |
| Massarina | Pearson's analysis | 0.93 | 1 | .976* | .960* | -0.826 | -0.722 | .998** | -0.905 |
|  | Significance | 0.07 |  | 0.024 | 0.04 | 0.174 | 0.278 | 0.002 | 0.095 |
| Thyridariaceae | Pearson's analysis | .987* | .976* | 1 | .994** | -0.901 | -0.84 | .963* | -0.924 |
|  | Significance | 0.013 | 0.024 |  | 0.006 | 0.099 | 0.16 | 0.037 | 0.076 |
| Entorrhiza | Pearson's analysis | .992** | .960* | .994** | 1 | -0.941 | -0.886 | 0.949 | -.954* |
|  | Significance | 0.008 | 0.04 | 0.006 |  | 0.059 | 0.114 | 0.051 | 0.046 |
| Penicillium | Pearson's analysis | 0.491 | 0.641 | 0.552 | 0.588 | -0.625 | -0.462 | 0.691 | -0.778 |
|  | Significance | 0.509 | 0.359 | 0.448 | 0.412 | 0.375 | 0.538 | 0.309 | 0.222 |
| Digitaria | Pearson's analysis | -0.938 | -0.826 | -0.901 | -0.941 | 1 | .977* | -0.82 | .966* |
|  | Significance | 0.062 | 0.174 | 0.099 | 0.059 |  | 0.023 | 0.18 | 0.034 |
| Acremonium | Pearson's analysis | -0.205 | -0.142 | -0.166 | -0.26 | 0.521 | 0.48 | -0.185 | 0.527 |
|  | Significance | 0.795 | 0.858 | 0.834 | 0.74 | 0.479 | 0.52 | 0.815 | 0.473 |
| Ascotricha | Pearson's analysis | -0.907 | -0.722 | -0.84 | -0.886 | .977* | 1 | -0.706 | 0.887 |
|  | Significance | 0.093 | 0.278 | 0.16 | 0.114 | 0.023 |  | 0.294 | 0.113 |
| Chaetomium | Pearson's analysis | 0.265 | -0.1 | 0.117 | 0.155 | -0.301 | -0.499 | -0.154 | -0.044 |
|  | Significance | 0.735 | 0.9 | 0.883 | 0.845 | 0.699 | 0.501 | 0.846 | 0.956 |
| Fusarium | Pearson's analysis | 0.516 | 0.709 | 0.617 | 0.532 | -0.216 | -0.11 | 0.693 | -0.342 |
|  | Significance | 0.484 | 0.291 | 0.383 | 0.468 | 0.784 | 0.89 | 0.307 | 0.658 |
| Nectria | Pearson's analysis | 0.593 | 0.256 | 0.459 | 0.51 | -0.663 | -0.809 | 0.213 | -0.446 |
|  | Significance | 0.407 | 0.744 | 0.541 | 0.49 | 0.337 | 0.191 | 0.787 | 0.554 |
| Ophiostoma | Pearson's analysis | -0.553 | -0.444 | -0.529 | -0.472 | 0.281 | 0.355 | -0.382 | 0.187 |
|  | Significance | 0.447 | 0.556 | 0.471 | 0.528 | 0.719 | 0.645 | 0.618 | 0.813 |
| Stephanonectria | Pearson's analysis | 0.911 | .998** | .963* | 0.949 | -0.82 | -0.706 | 1 | -0.913 |
|  | Significance | 0.089 | 0.002 | 0.037 | 0.051 | 0.18 | 0.294 |  | 0.087 |
| Trichoderma | Pearson's analysis | -0.921 | -0.905 | -0.924 | -.954* | .966* | 0.887 | -0.913 | 1 |
|  | Significance | 0.079 | 0.095 | 0.076 | 0.046 | 0.034 | 0.113 | 0.087 |  |

**Supplementary Figures**


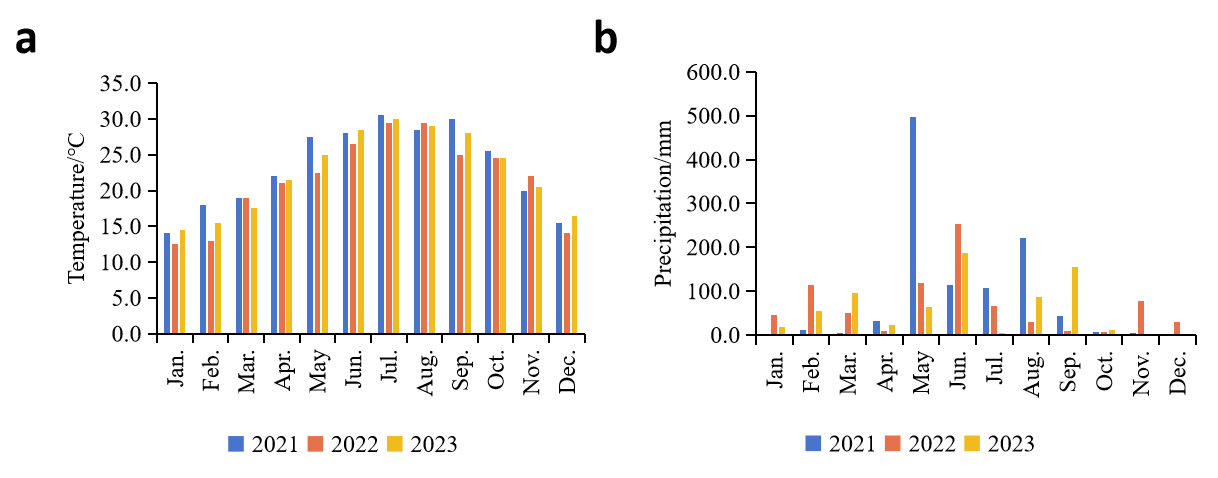


**^Fig. S1^** ^Meteorological conditions: temperature (a) and precipitation (b) during experimental period (2021–2023).^


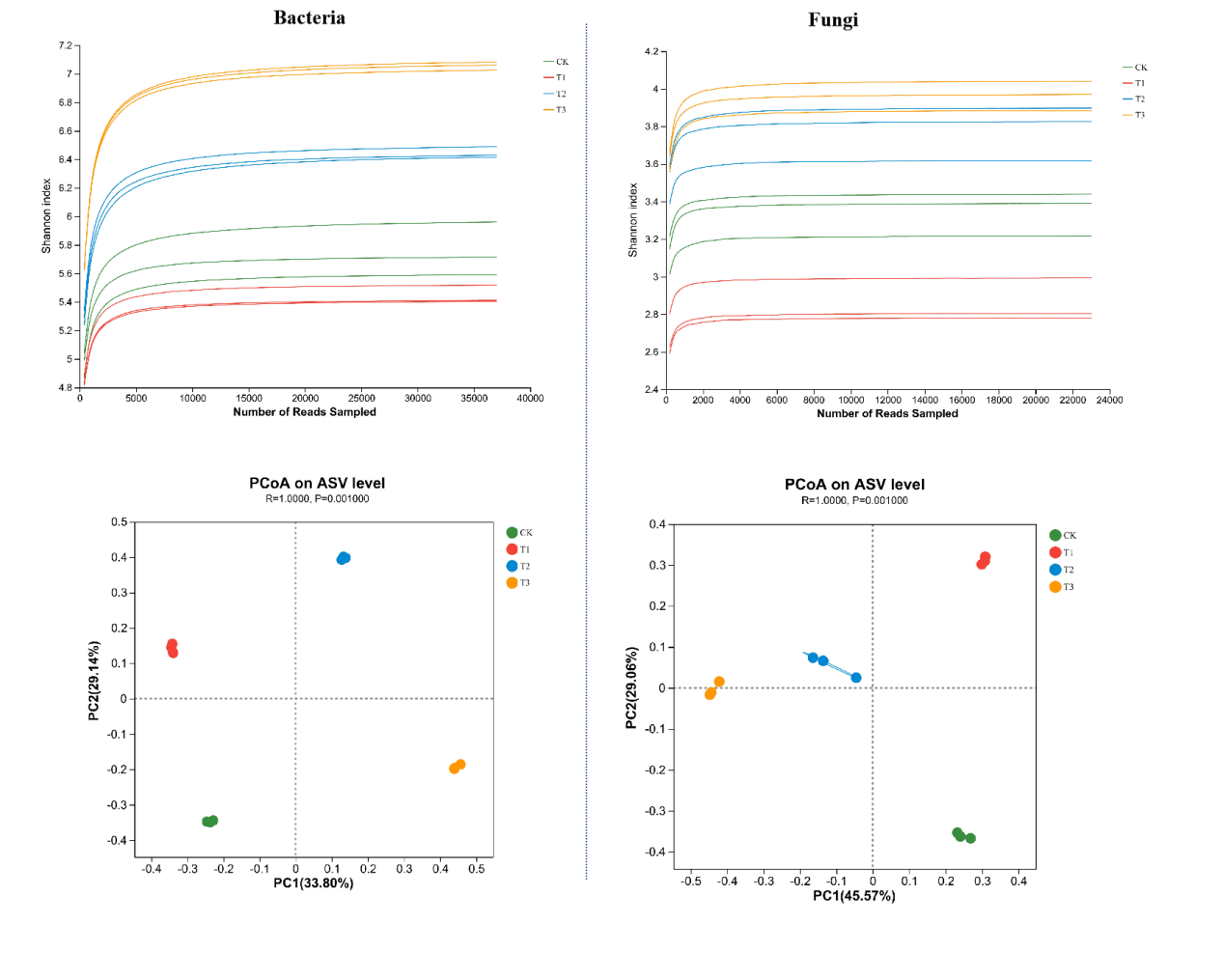


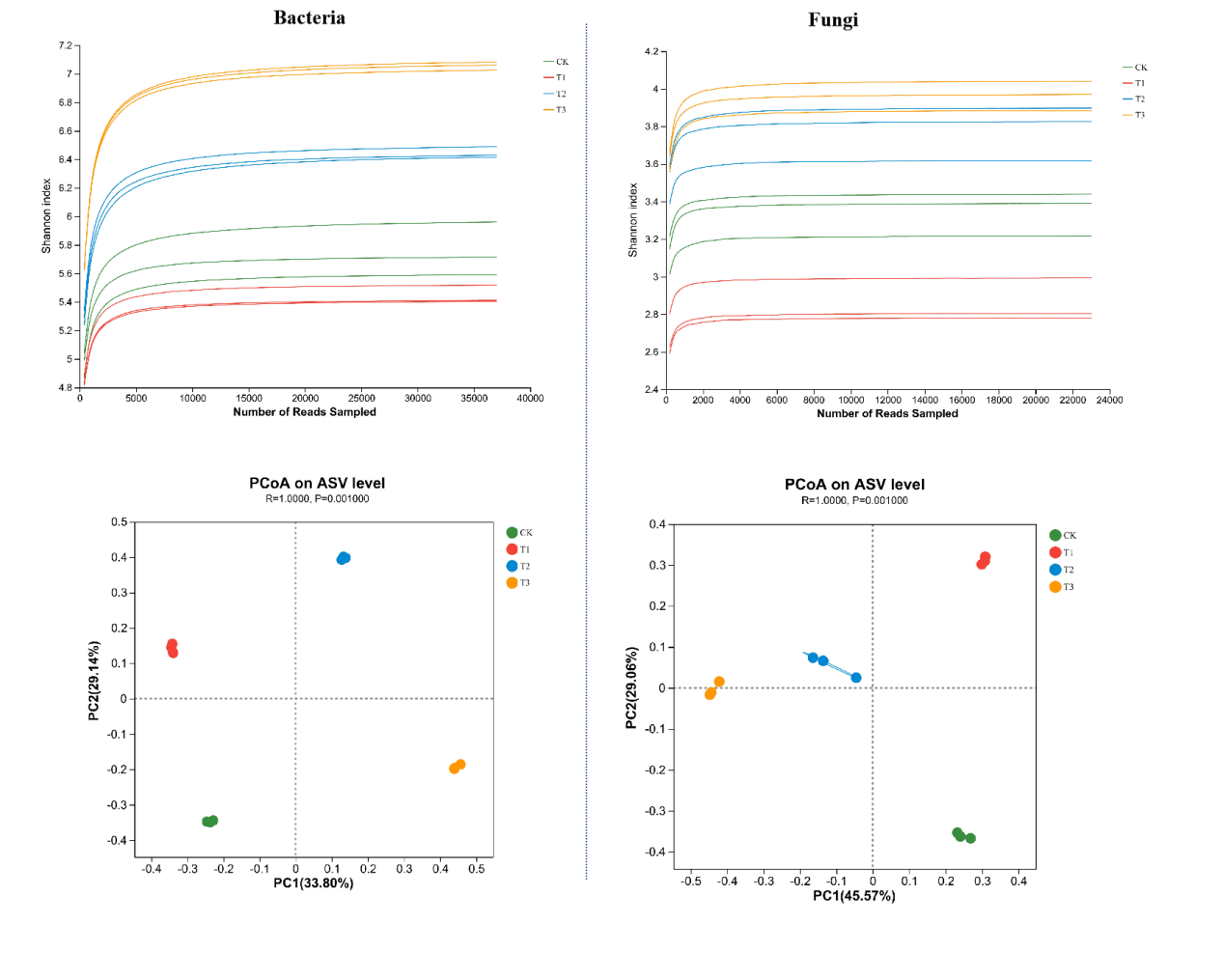
**Fig. S2** Rarefaction curves and PCoA analysis of bacterial community and fungal community


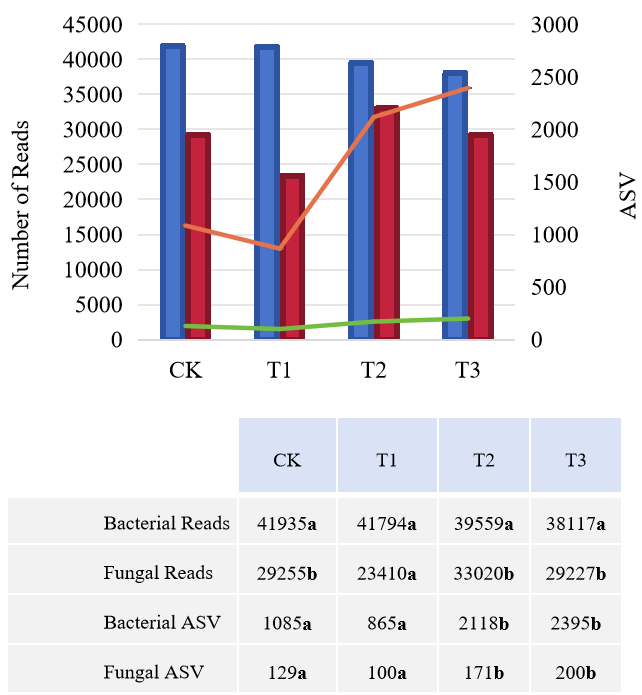


**Fig. S3** The analysis of ASV distribution
